# Supplementary material for: MET inhibition overcomes radiation resistance of glioblastoma stem‐like cells
Source: EMBO Mol Med. 2016 Apr 4;8(5):550–68. doi: 10.15252/emmm.201505890 (PMC5130292; doi:10.15252/emmm.201505890)
Supplement: Supplementary file 1 — Appendix [file EMMM-8-550-s001.pdf]

MET inhibition overcomes radiation resistance of glioblastoma stem-like cells

Francesca De Bacco[1], Antonio D'Ambrosio[1,2], Elena Casanova[1], Francesca Orzan[1], Roberta Neggia[1], Raffaella Albano[1], Federica Verginelli[1], Manuela Cominelli[3], Pietro L. Poliani[3], Paolo Luraghi[1], Gigliola Reato[1,2], Serena Pellegatta[4], Gaetano Finocchiaro[4], Timothy Perera[5], Elisabetta Garibaldi[6], Pietro Gabriele[6], Paolo M. Comoglio[1,2,7] and Carla Boccaccio[1,2,7]

Table of contents:

- Appendix Tables S1-5

- Appendix Figures S1-9

- Appendix Methods

- Appendix References

Appendix Tables

Table S1. Clinical data of primary glioblastomas (WHO grade IV gliomas) originating neurospheres.

Patient code	Gender	Age	DFS (months)	OS (months)	Location
**BT205**	M	73	0	2	T dx
**BT273**	M	58	n.a.	12	FTP dx
**BT275**	F	66	n.a.	14	P dx
**BT287**	M	70	14	16	T sx
**BT302**	F	17	n.a.	60	F dx
**BT308**	M	76	0	3	T sx
**BT314**	M	67	n.a.	18	T sx
**BT328**	M	54	0	6	FP sx
**BT334**	M	63	n.a.	9	FT dx
**BT337**	M	63	9.5	17.5	FTP sx
**BT347**	F	54	25	29	T sx
**BT358**	F	63	n.a.	7.5	F dx
**BT371**	F	46	4	14	F sx
**BT373**	M	62	9	22	F dx
**BT398**	M	48	4	8	T sx
**BT421**	M	66	n.a.	15	T dx
**BT437**	M	49	10	12	T sx
**BT443**	M	65	5.5	19	T sx
**BT452**	F	66	0	0	T dx
**BT463**	M	69	4	11	TP sx

DFS: Disease Free Survival (months); OS: Overall Survival (months); n.a.: not available.
Location: T (temporal); F (frontal); P (parietal).

Table S2. Genetic mutations and expression profile of neurospheres (NS)

NS	EGFR[amp]	PTEN[loss]	TP53[mut]	NFKBIA[del]	GEP subtype[a]
**BT205**	+	+	+	-	CL
**BT273**	+	-	+	-	CL
**BT275**	+	-	+	-	CL
**BT287**	-	+	+	n.a.	MES
**BT302**	-	+	-	-	MES
**BT308**	-	+	+	+	PN
**BT314**	-	+	+	+	PN
**BT328**	-	+	+	+	CL
**BT334**	+	-	-	-	CL
**BT337**	-	-	-	+	PN
**BT347**	-	+	+	-	MES
**BT358**	+	-	-	+	MES
**BT371**	-	+	+	+	MES
**BT373**	+	-	-	+	CL/PN
**BT398**	-	+	-	+	CL
**BT421**	-	+	+	+	CL
**BT437**	-	+	-	+	CL
**BT443**	-	-	+	+	CL
**BT452**	+	+	+	+	MES
**BT463**	-	-	+	-	PN

+: presence of genetic lesions; -: absence of genetic lesions; loss: homozygous deletion or hemizygous mutation;
amp: copy number difference between EGFR and HGF > 5; del: copy number < 1.5; mut: mutation; n.a.: not available.
a: gene expression profile (GEP) subtype according to the Verhaak's signature (Verhaak *et al.*, 2010); CL: classical; MES: mesenchymal; PN: proneural.

Table S3. MET/HGF expression in neurospheres (NS)

	NS	MET mRNA[a]	MET protein (% pos cells)[b]	HGF mRNA[a]	HGF protein (a.u.)[c]
MET-pos-NS	**BT205**	5.9	5.1 ± 0.1	3.11	8.8
	**BT287**	12.7	64.22 ± 0.28	11.66	n.a.
	**BT302**	7.4	17 ± 2	4.6	2.4
	**BT308**	13.7	46 ± 2	3.26	5.2
	**BT314**	3.1	15.5 ± 0.5	11.09	3.4
	**BT328**	12.6	16.77 ± 4.77	3.02	n.a.
	**BT337**	12.5	92 ± 2	neg	0.8
	**BT347**	9.5	17.75 ± 1.25	6.497	n.a.
	**BT371**	15.4	90.5 ± 8.5	7.6	2
	**BT398**	11	93.04 ± 1.045	6.75	n.a.
	**BT437**	8.4	17.91 ± 3.03	4.21	n.a.
	**BT452**	13.8	95.45 ± 0.85	3.77	3.3
	**BT463**	10.6	70 ± 0	2.97	5.2
MET-neg-NS	**BT273**	2.6	neg	neg	neg
	**BT275**	1.9	neg	neg	neg
	**BT334**	4.2	neg	neg	neg
	**BT358**	0.9	neg	neg	neg
	**BT373**	1.6	neg	neg	neg
	**BT421**	8.6	neg	neg	neg
	**BT443**	2.8	neg	neg	neg

a: MET/HGF mRNA measured by real time PCR and reported as 40-Ct;
b: MET protein measured by flow-cytometry. Percentage of positive cells was reported as mean ± SEM.
c: HGF protein measured by western blot on NS lysates. HGF densitometric quantification was normalized on β-actin.
a.u.: arbitrary units. n.a.: not available.

Table S4. Clinical data of matched primary and recurrent glioblastomas (WHO grade IV gliomas).

Patient	Gender	Age	Location	RTOG-RPA	1st surgery	MGMT	Adjuvant treatment after 1st surgery	DFS (months)	OS (months)
**1**	F	25	T	III	Partial	NM	RT+CT	1	10
**2**	M	56	N	IV	Total/subtotal	M	RT+CT	4	22
**3**	F	59	P	IV	Partial	NM	RT+CT	34	50
**4**	M	63	T	IV	Total/subtotal	NM	RT+CT	14	27
**5**	M	45	F	III	Partial	n.a.	RT+CT	15	26
**6**	M	65	P	IV	Total/subtotal	n.a.	RT+CT	6	8
**7**	M	65	P	V	Total/subtotal	n.a.	RT+CT	10	23
**8**	M	47	T	III	Total/subtotal	n.a.	RT+CT	23	32
**9**	M	65	T	IV	Total/subtotal	NM	RT+CT	17	25
**10**	M	50	T	V	Total/subtotal	NM	RT+CT	11	21
**11**	F	39	F	III	Partial	NM	RT+CT	4	10
**12**	F	62	P	IV	Total/subtotal	NM	RT+CT	7	9
**13**	M	52	T	IV	Total/subtotal	M	RT+CT	17	42
**14**	M	64	F	III	Total/subtotal	NM	RT+CT	11	15
**15**	M	51	T	V	Total/subtotal	NM	RT+CT	4	16
**16**	M	55	F	IV	Total/subtotal	NM	RT+CT	10	15
**17**	F	48	F	I	Total/subtotal	M	RT+CT	12	19
**18**	F	54	T	V	Total/subtotal	NM	RT+CT	17	22
**19**	M	68	T	IV	Total/subtotal	NM	RT+CT	7	18

Location: T (temporal); F (frontal); P (parietal); N (basal nuclei). RTOG-RPA: Radiation Therapy Oncology Group recursive partitioning analysis. MGMT: NM (non-methylated); M (methylated); n.a. (not available). Treatment: RT, radiotherapy; CT, chemotherapy (Temozolomide). DSF: Disease Free Survival; OS: Overall Survival.

Table S5. MET expression (immunohistochemistry) in primary and recurrent human GBMs.

Patient	Primary GBM			Recurrent GBM		
	MET positive cells (score)[a]	MET staining intensity (score)[b]	MET cumulative score (a+b)	MET positive cells (score)[a]	MET staining intensity (score)[b]	MET cumulative score (a+b)
**1**	0	1	1	3	3	6
**2**	1	1	2	2	2	4
**3**	0	0	0	1	1	2
**4**	0	1	1	0	1	1
**5**	0	1	1	1	1	2
**6**	0	1	1	0	1	1
**7**	1	1	2	1	1	2
**8**	0	0	0	2	1	3
**9**	1	1	2	1	1	2
**10**	3	1	4	3	1	4
**11**	0	0	0	3	2	5
**12**	3	3	6	3	3	6
**13**	0	0	0	0	1	1
**14**	2	2	4	3	2	5
**15**	1	1	2	1	1	2
**16**	0	1	1	1	1	2
**17**	0	1	1	2	2	4
**18**	0	1	1	2	1	3
**19**	0	1	1	1	1	2

a: positivity scores: 0, 0-5% cells; 1, 6-29% cells; 2, 30-69% cells; 3, 70-100% cells.
b: staining intensity scores: 0, negative; 1, low; 2, moderate; 3, high.

Appendix Figures

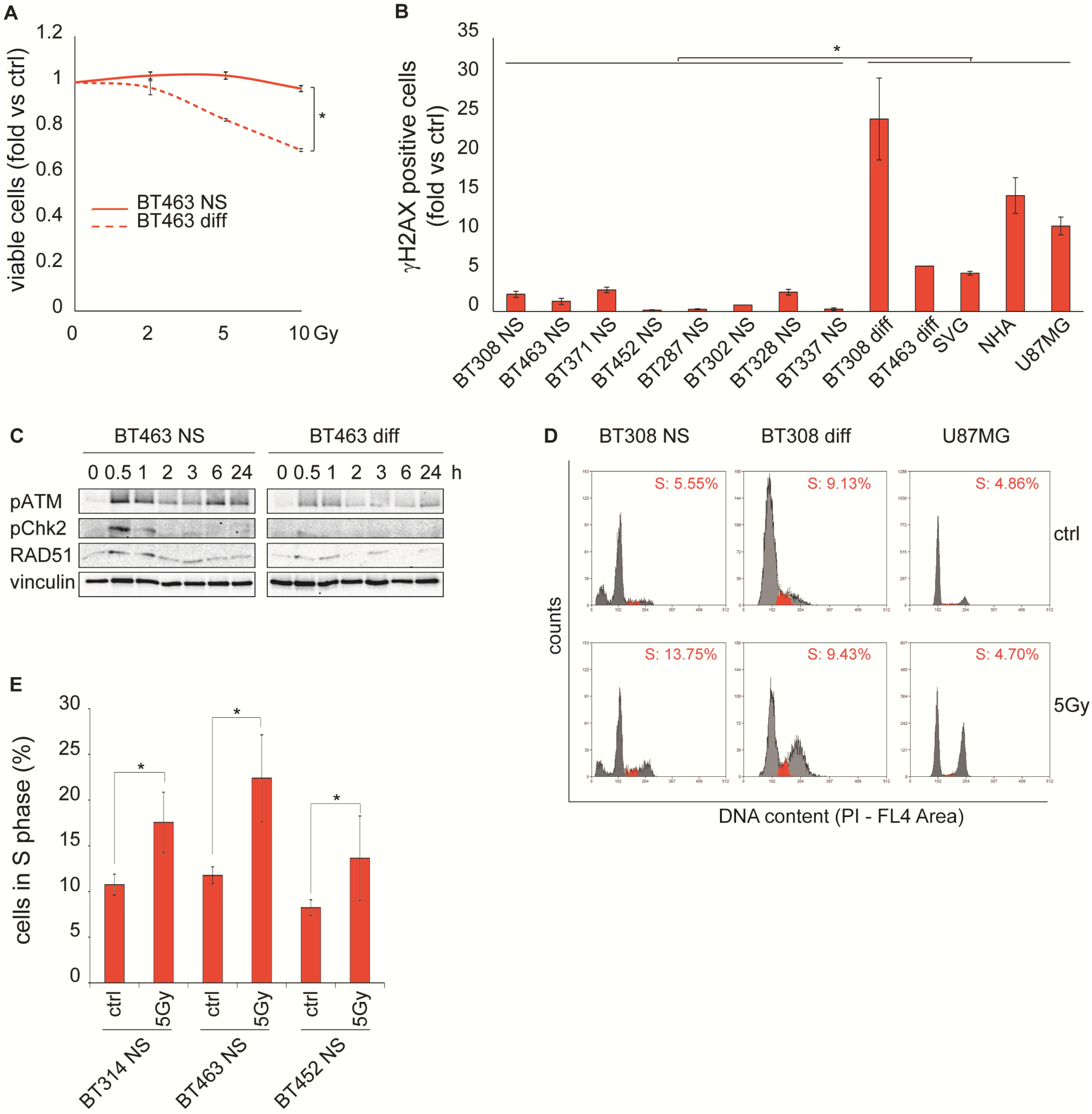

F

BT463	ctrl	5Gy
sub G_0	2.19 ± 0.34	1.81 ± 0.12
G_0 - G_1	74.10 ± 5.63	56.95 ± 4.47
S	11.76 ± 0.93	22.4 ± 4.74
G_2 - M	12.56 ± 5.36	22.7 ± 2.21

BT314	ctrl	5Gy
sub G_0	5.86 ± 1.47	9.98 ± 2.65
G_0 - G_1	72.06 ± 1.9	52.34 ± 2.63
S	10.75 ± 1.13	17.56 ± 3.29
G_2 - M	12.34 ± 0.54	20.5 ± 2.64

BT452	ctrl	5Gy
sub G_0	5.51 ± 4.56	3.19 ± 2.21
G_0 - G_1	78.67 ± 6.93	71.68 ± 4.8
S	8.23 ± 0.85	13.64 ± 4.62
G_2 - M	7.88 ± 1.21	12.18 ± 2.73

Appendix Fig S1. GSCs are radioresistant.

A, Cell viability of BT463 NS and their pseudodifferentiated progeny (BT463 diff) measured 24 h after irradiation (fold vs. non-irradiated cells, ctrl). Data are represented as mean ± SEM. *: *t*-test, $p<0.0001$. Overlapping results were obtained with other NS (data not shown). B, Flow cytometric analysis of phosphorylated histone H2AX (γH2AX) 6 h after IR (5 Gy, fold vs. non-irradiated cells, ctrl). Data are represented as mean ± SEM. *: one-way ANOVA test, $p<0.01$. C, Western blot on BT463 NS and their pseudodifferentiated progeny (BT463 diff) showing phosphorylation of ATM (pATM) and Chk2 (pChk2), and accumulation of RAD51 after IR (5Gy). Vinculin was used as loading control. D, Flow-cytometric analysis of cell cycle 24 h after IR (5 Gy). The percentage of S phase-cells (red) is indicated. Ctrl: non-irradiated cells. E, Percentage of cells in S phase measured by flow cytometric analysis of cell cycle 24 h after IR (5 Gy). Data are represented as mean ± SEM. Ctrl: non-irradiated cells. *: one-way ANOVA test (S phase), $P=0.01$. F, Tables showing the percentage of cells in different phases of cell cycle measured in flow cytometry 24 h after IR (5 Gy). Data are mean ± SEM. Ctrl: non-irradiated cells.

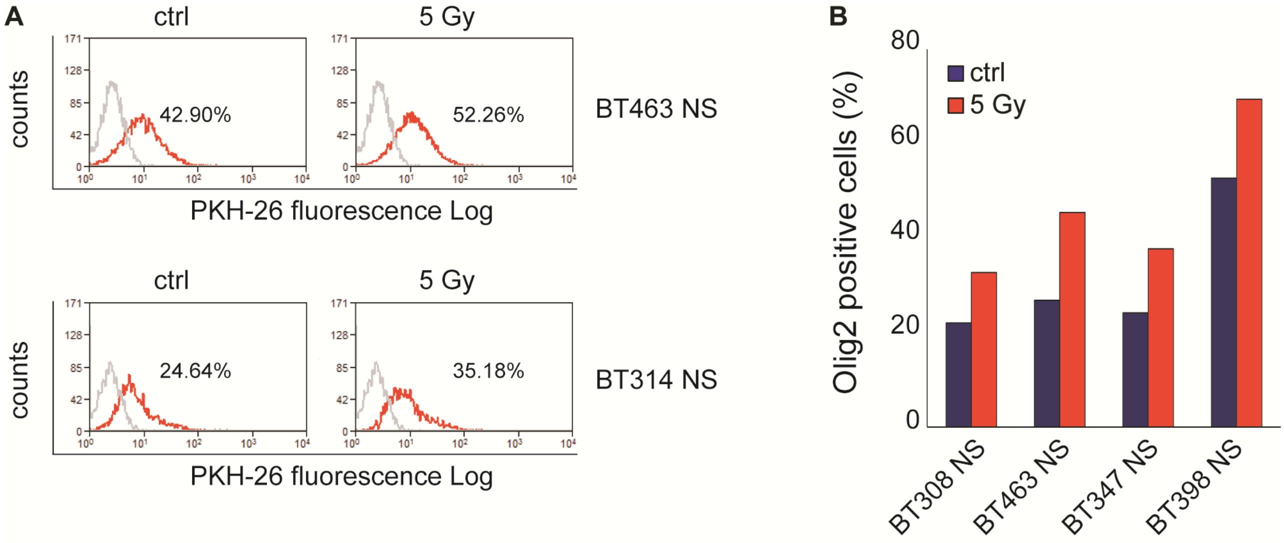

Appendix Fig S2. GSCs are positively selected by IR.

A, Flow cytometric analysis of PKH-26 staining in irradiated BT463 NS and BT314 NS, measured 24 h after IR (5 Gy). The percentage of PKH-26^{pos} cells is indicated. Ctrl: non-irradiated cells. B, Flow-cytometric detection of Olig2 in NS 24 h after IR (5 Gy).

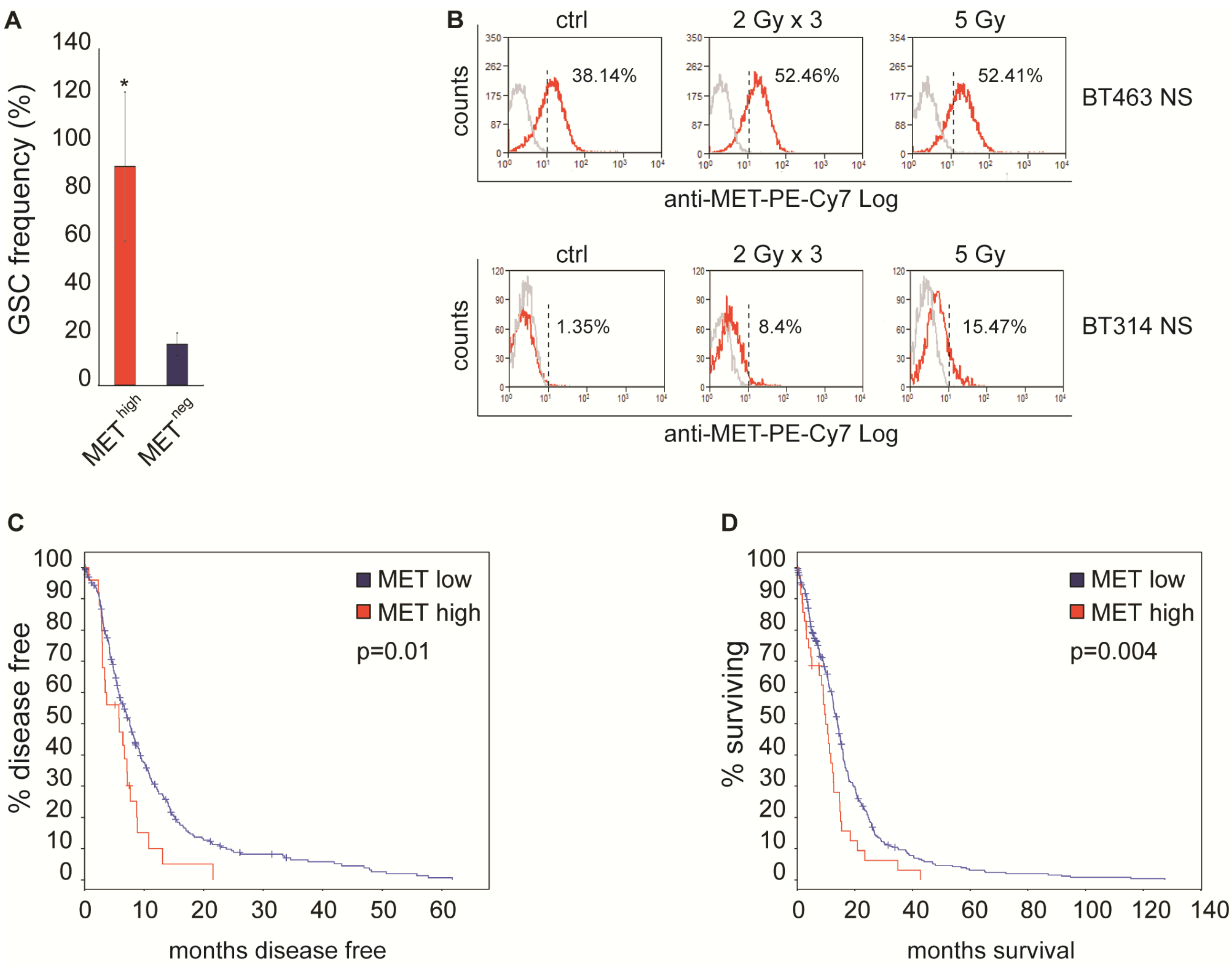

Appendix Fig S3. MET-expressing GSC are enriched after NS irradiation.

A, Limiting dilution sphere forming assay measuring the frequency of GSC after IR (5 Gy) in MET^{high} and MET^{neg} subpopulation sorted from BT452 NS. Data are represented as mean ± CI. *: χ^2 test, P=2.85×10^{-5}. B, Flow cytometric analysis of MET in irradiated BT463 NS and BT314 NS, 24 h after the last irradiation (2 Gy × 3 days or a single dose of 5 Gy). Dotted line: threshold to define the percentage of MET positive cells. Ctrl: non-irradiated cells. The MFI was as follow: BT463 NS *ctrl*, 25.81; *2 Gy* × *3*, 27.74; *5 Gy*, 28.76; BT314 NS *ctrl*, 16.12; *2 Gy* × *3*, 29.09; *5 Gy*, 20.38. C, Disease-free survival curve of primary GBM patients included in the TCGA provisional dataset (June 2015). Red line: patients expressing MET mRNA levels > (mean + 2 × SD) (n = 25). Blue line: all other patients (n = 271). P values from log-rank test were indicated. D, Survival curve of patients defined as in (C) (red line n = 35; blue line, n = 333). P values from log-rank test were indicated.

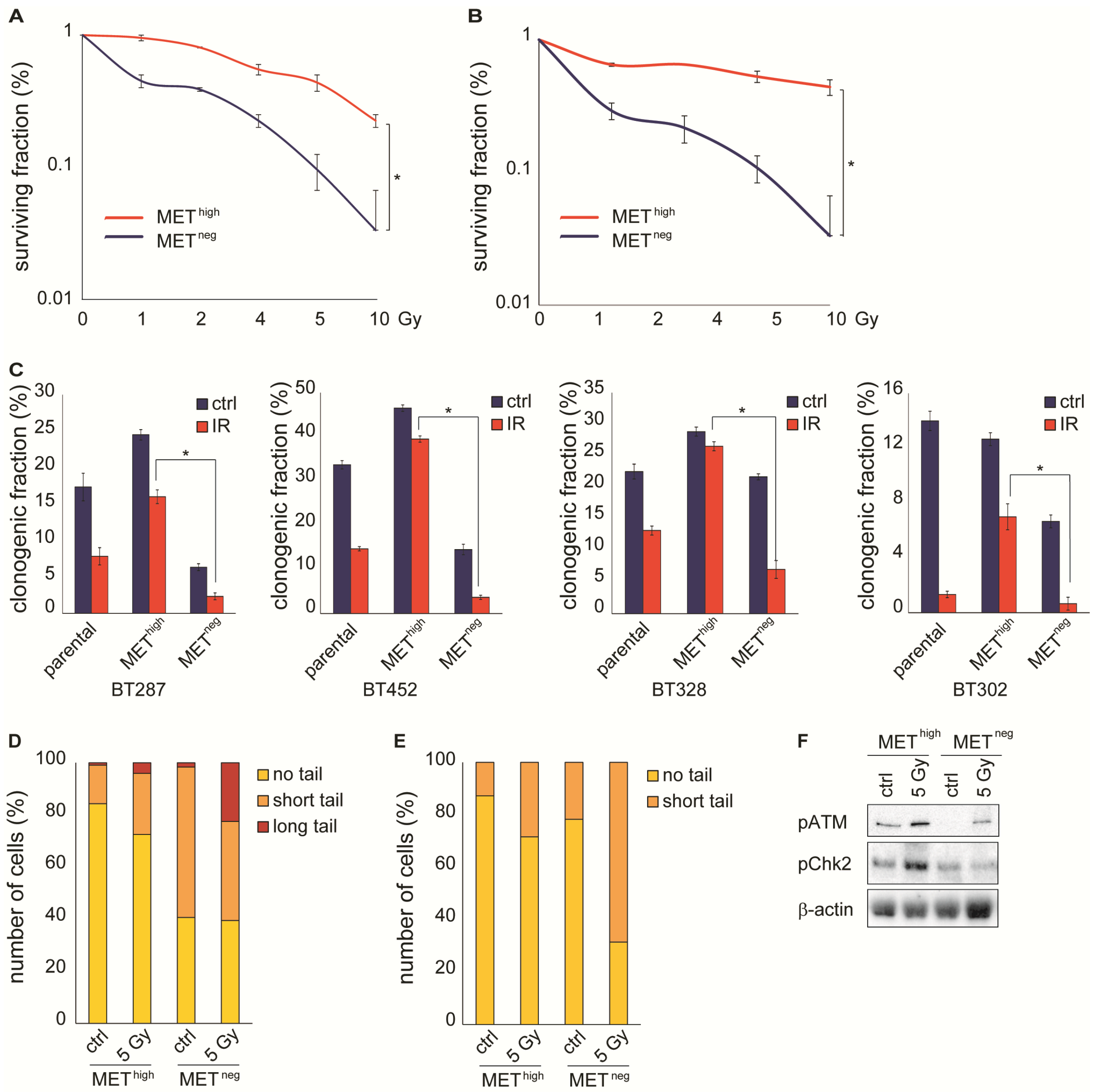

Appendix Fig S4. The MET^{high} GSC subpopulation efficiently activates DDR.

A-B, Clonogenic assay with MET^{high} and MET^{neg} subpopulations sorted from BT463 NS (A) or BT205 NS (B) and irradiated (1-10 Gy, fold vs. non-irradiated cells). Data are represented as mean ± SEM. *: *t*-test, $p<0.05$. C, Clonogenic assay with MET^{high} and MET^{neg} subpopulations sorted from BT287, BT452, BT328 or BT302 neurospheres and irradiated (5 Gy, fold vs. non-irradiated cells). Data are represented as mean ± SEM. *: *t*-test, $p<0.05$. D-E, Neutral comet assay in MET^{high} and MET^{neg} subpopulations sorted from BT463 NS (D) or BT205 NS (E), performed 24 h after IR (5 Gy). Ctrl: non-irradiated cells. Comet tail's length is proportional to DSB extent. F, Western blot of MET^{high} and MET^{neg} subpopulations sorted from BT452 NS, showing constitutive (ctrl) and/or

IR-induced phosphorylation of ATM (pATM) and Chk2 (pChk2) 24 h after IR (5 Gy). β-actin was used as loading control.

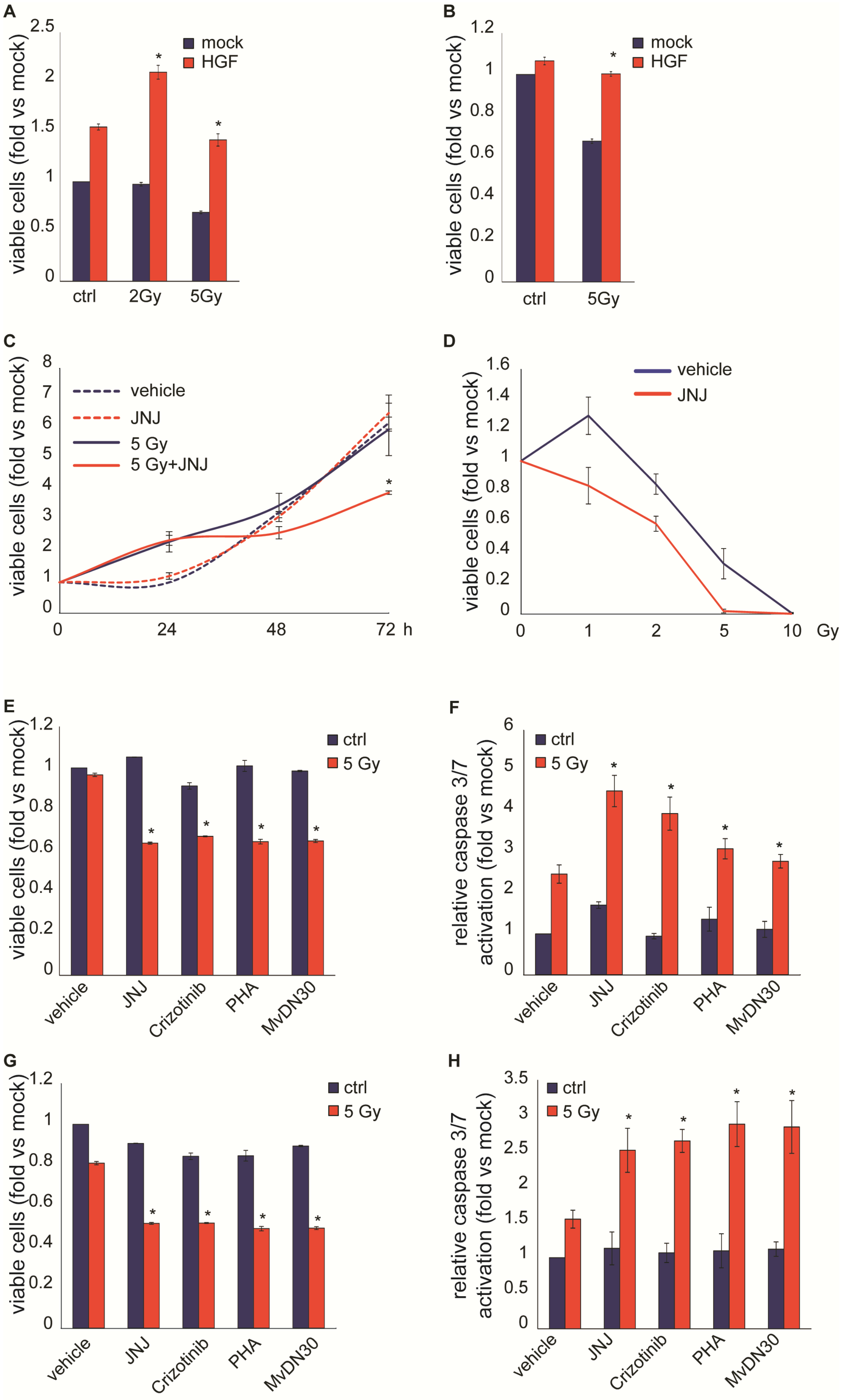
A
viable cells (fold vs mock)
2.5
2
1.5
1
0.5
0
mock
HGF
*
*
ctrl
2Gy
5Gy
B
viable cells (fold vs mock)
1.2
1
0.8
0.6
0.4
0.2
0
mock
HGF
*
ctrl
5Gy
C
viable cells (fold vs mock)
8
7
6
5
4
3
2
1
0
vehicle
JNJ
5 Gy
5 Gy+JNJ
*
0
24
48
72 h
D
viable cells (fold vs mock)
1.6
1.4
1.2
1
0.8
0.6
0.4
0.2
0
vehicle
JNJ
0
1
2
5
10 Gy
E
viable cells (fold vs mock)
1.2
1
0.8
0.6
0.4
0.2
0
ctrl
5 Gy
*
*
*
*
vehicle
JNJ
Crizotinib
PHA
MvDN30
F
relative caspase 3/7 activation (fold vs mock)
6
5
4
3
2
1
0
ctrl
5 Gy
*
*
*
*
vehicle
JNJ
Crizotinib
PHA
MvDN30
G
viable cells (fold vs mock)
1.2
1
0.8
0.6
0.4
0.2
0
ctrl
5 Gy
*
*
*
*
vehicle
JNJ
Crizotinib
PHA
MvDN30
H
relative caspase 3/7 activation (fold vs mock)
3.5
3
2.5
2
1.5
1
0.5
0
ctrl
5 Gy
*
*
*
*
vehicle
JNJ
Crizotinib
PHA
MvDN30

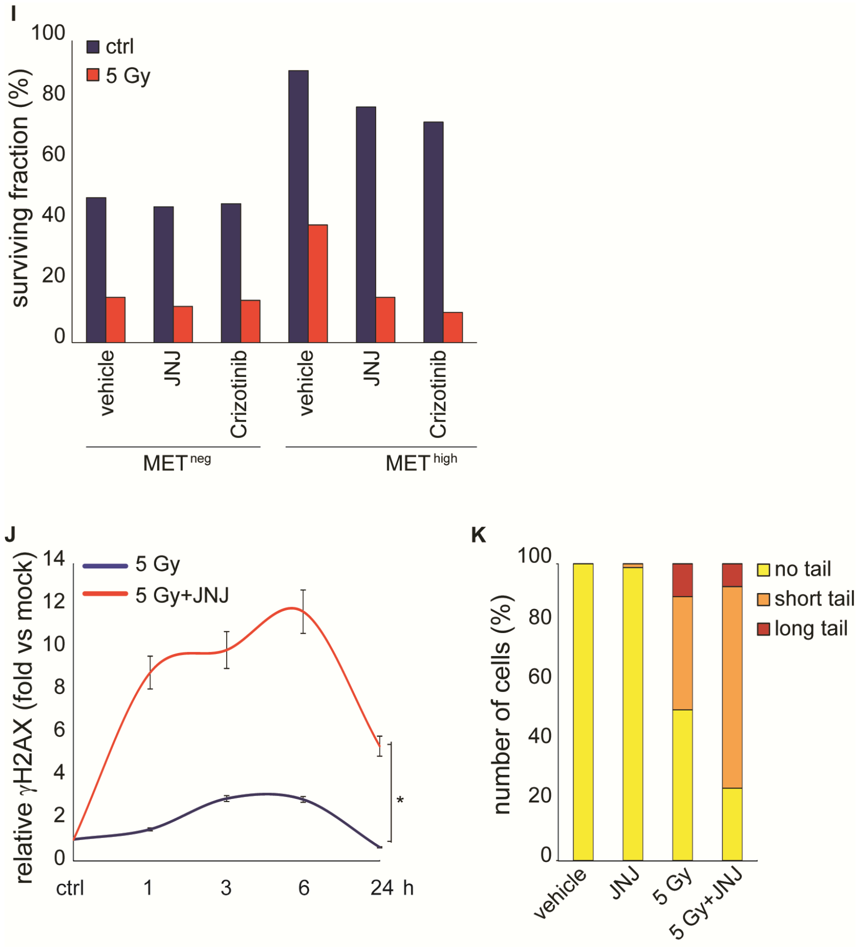

Appendix Fig S5. MET inhibition radiosensitizes GSCs.

A-B, Cell viability of BT463 NS (A) or BT205 NS (B), measured 72 h or 48 h after IR (5 Gy), respectively, in the absence (mock) or in the presence of HGF (fold vs. non-irradiated cells, mock). Data are represented as mean ± SEM. *: *t*-test (5 Gy + HGF) vs. (5 Gy), p<0.0001. C, Cell viability of BT463 NS, irradiated in the absence (5 Gy) or in the presence (5 Gy + JNJ) of the MET inhibitor JNJ38877605, and analyzed at the indicated time points. Vehicle: non-irradiated cells (fold vs. vehicle-treated cells at time 0, mock). Data are represented as mean ± SEM. *: *t*-test, p<0.05. Overlapping results were obtained with BT371 NS (data not shown). D, Cell viability as in (C), measured 5 days after IR (1-10 Gy, fold vs. vehicle-treated cells, mock). Data are represented as mean ± SEM. *: *t*-test, p<0.05. Overlapping results were obtained with BT371 NS (data not shown). E, Cell viability of BT463 NS, measured 72 h after IR (5 Gy) in the absence (vehicle) or in the presence of MET inhibitors (JNJ38877605, Crizotinib, PHA665752) or the MET antibody MvDN30 (fold vs. non-irradiated vehicle-treated cells, mock). Data are represented as mean ± SEM. *: *t*-test (5 Gy + inhibitor) vs. (5 Gy + vehicle), p<0.05. Overlapping results were obtained also with BT371 NS and BT452 NS (data not shown). F, Analysis of caspase 3/7 activation in BT463 NS, performed 48 h after IR (5 Gy) as in (E) (fold vs. non-irradiated vehicle-treated cells, mock). Data are represented as mean ± SEM. *: *t*-test (5 Gy + inhibitor) vs. (5 Gy + vehicle),

$p<0.05$. Overlapping results were obtained with BT371 NS and BT452 NS (data not shown). G, Cell viability of BT308 NS, measured 48 h after IR (5 Gy) in the absence (vehicle) or in the presence of MET inhibitors (JNJ38877605, Crizotinib, PHA665752) or the MET antibody MvDN30 (fold vs. non-irradiated vehicle-treated cells, mock). Data are represented as mean ± SEM. *: *t*-test (5 Gy + inhibitor) vs. (5 Gy + vehicle), $p<0.05$. H, Analysis of caspase 3/7 activation in BT308 NS, performed 24 h after IR (5 Gy) as in (G) (fold vs. non-irradiated vehicle-treated cells, mock). Data are represented as mean ± SEM. *: *t*-test (5 Gy + inhibitor) vs. (5 Gy + vehicle), $p<0.05$. I, Clonogenic assay with MET^{neg} and MET^{high} subpopulations sorted from BT463 NS, irradiated (5 Gy) in the absence (vehicle) or in the presence of MET inhibitors (JNJ38877605 and Crizotinib). Ctrl: non-irradiated cells. *: *t*-test, $p<0.0001$. J, Flow cytometric analysis of the amount of phosphorylated histone H2AX (γH2AX) in BT371 NS, measured at the indicated time points after IR in the absence (5 Gy) or in the presence (5Gy + JNJ) of JNJ38877605 (fold vs. non-irradiated cells at time 0, mock). Data are represented as mean ± SEM. *: *t*-test, $p<0.05$. K, Neutral comet assay 24 h after treatment of BT463 NS with IR in the absence (5 Gy) or in the presence (5 Gy + JNJ) of JNJ38877605.

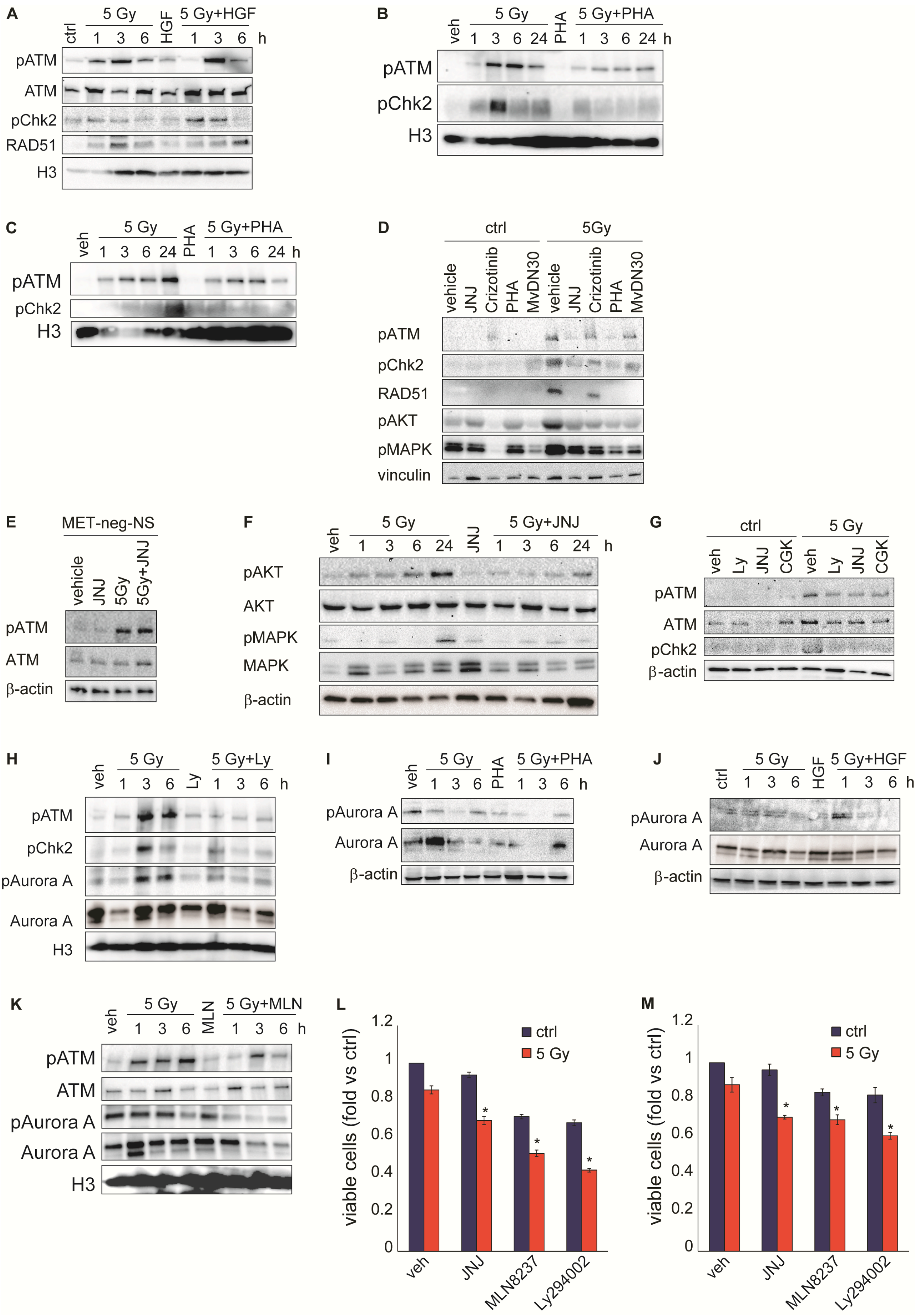
A
ctrl
5 Gy
HGF
5 Gy+HGF
1 3 6 1 3 6 h
pATM
ATM
pChk2
RAD51
H3
B
veh
5 Gy
PHA
5 Gy+PHA
1 3 6 24 1 3 6 24 h
pATM
pChk2
H3
C
veh
5 Gy
PHA
5 Gy+PHA
1 3 6 24 1 3 6 24 h
pATM
pChk2
H3
D
ctrl
5Gy
vehicle
JNJ
Crizotinib
PHA
MvDN30
pATM
pChk2
RAD51
pAKT
pMAPK
vinculin
E
MET-neg-NS
vehicle
JNJ
5Gy
5Gy+JNJ
pATM
ATM
β-actin
F
veh
5 Gy
JNJ
5 Gy+JNJ
1 3 6 24 1 3 6 24 h
pAKT
AKT
pMAPK
MAPK
β-actin
G
ctrl
5 Gy
veh
Ly
JNJ
CGK
pATM
ATM
pChk2
β-actin
H
veh
5 Gy
Ly
5 Gy+Ly
1 3 6 1 3 6 h
pATM
pChk2
pAurora A
Aurora A
H3
I
veh
5 Gy
PHA
5 Gy+PHA
1 3 6 1 3 6 h
pAurora A
Aurora A
β-actin
J
ctrl
5 Gy
HGF
5 Gy+HGF
1 3 6 1 3 6 h
pAurora A
Aurora A
β-actin
K
veh
5 Gy
MLN
5 Gy+MLN
1 3 6 1 3 6 h
pATM
ATM
pAurora A
Aurora A
H3
L
viable cells (fold vs ctrl)
1.2
1
0.8
0.6
0.4
0.2
0
ctrl
5 Gy
veh
JNJ
MLN8237
Ly294002
M
viable cells (fold vs ctrl)
1.2
1
0.8
0.6
0.4
0.2
0
ctrl
5 Gy
veh
JNJ
MLN8237
Ly294002

Appendix Fig S6. MET inhibition impairs AKT-dependent ATM activity.

A, Western blot of BT308 NS showing phosphorylation of ATM (pATM) and Chk2 (pChk2), and accumulation of RAD51 at the indicated time points after IR in the absence (5 Gy) or in the presence (5 Gy + HGF) of HGF. Total ATM is also shown. H3 was used as loading control. B-C, Western blot of BT308 NS (B) or BT463 NS (C) showing phosphorylation of ATM and Chk2 at the indicated time points after IR in the absence (5 Gy) or in the presence (5 Gy + PHA) of the MET inhibitor PHA665752. H3 was used as loading control. Veh: non-irradiated vehicle treated cells. D, Western blot of BT308 NS showing phosphorylation of ATM, Chk2, AKT (pAKT) and MAPK (pMAPK), and accumulation of RAD51, 24 h after IR in the absence (vehicle) or in the presence of MET inhibitors (JNJ38877605, Crizotinib, PHA665752, and MvDN30). Vinculin was used as loading control. Ctrl: non-irradiated cells. E, Western blot of BT373 NS showing phosphorylation of ATM 24 h after IR (5 Gy) in the absence (vehicle) or in the presence (5 Gy + JNJ) of the MET inhibitor JNJ38877605. Total ATM is also shown. β-actin was used as loading control. F, Western blot of BT308 NS showing phosphorylation of AKT and MAPK at the indicated time points after IR in the absence (5 Gy) or in the presence (5 Gy + JNJ) of JNJ38877605. Total AKT and MAPK are also shown. β-actin was used as loading control. Veh: non-irradiated vehicle treated cells. G, Western blot of BT463 NS showing phosphorylation of ATM and Chk2 24 h after IR (5 Gy) in the absence (vehicle, veh) or in the presence of AKT (Ly294002), MET (JNJ38877605), or ATM (CGK733) inhibitors. Total ATM is also shown. β-actin was used as loading control. Ctrl: non-irradiated cells. H, Western blot of BT308 NS showing phosphorylation of ATM, Chk2, and Aurora Kinase A (pAurora A) at the indicated time points after IR in the absence (5 Gy) or in the presence (5 Gy + Ly) of the AKT inhibitor Ly294002. Total Aurora Kinase A is also shown. H3 was used as loading control. Veh: non-irradiated vehicle-treated cells. I, Western blot of BT308 NS showing phosphorylation of Aurora Kinase A at the indicated time points after IR in the absence (5 Gy) or in the presence (5 Gy + PHA) of PHA665752. Total Aurora kinase A is also shown. β-actin was used as loading control. Veh: non-irradiated vehicle-treated cells. J, Western blot of BT308 NS showing phosphorylation of Aurora Kinase A at the indicated time points after IR in the absence (5 Gy) or in the presence (5 Gy + HGF) of HGF. Total Aurora kinase A is also shown. β-actin was used as loading control. K, Western blot of BT463 NS showing phosphorylation of ATM and Aurora Kinase A at the indicated time points after IR in the absence (5 Gy) or in the presence (5 Gy + MLN) of the Aurora Kinase A inhibitor MLN8237. Total ATM and Aurora kinase A are also shown. H3 was used as loading control. Veh: non-irradiated vehicle-treated cells. L-M, Cell viability of BT463 NS (L) or BT205 NS (M), measured 72 h after IR (5 Gy), in the absence (vehicle) or in the presence of MET (JNJ38877605), Aurora Kinase A (MLN8237), or AKT

(Ly294002) inhibitors (fold vs. non-irradiated vehicle-treated cells, mock). *: *t*-test (5 Gy + inhibitor) vs. (5 Gy + vehicle), $P<0.01$.

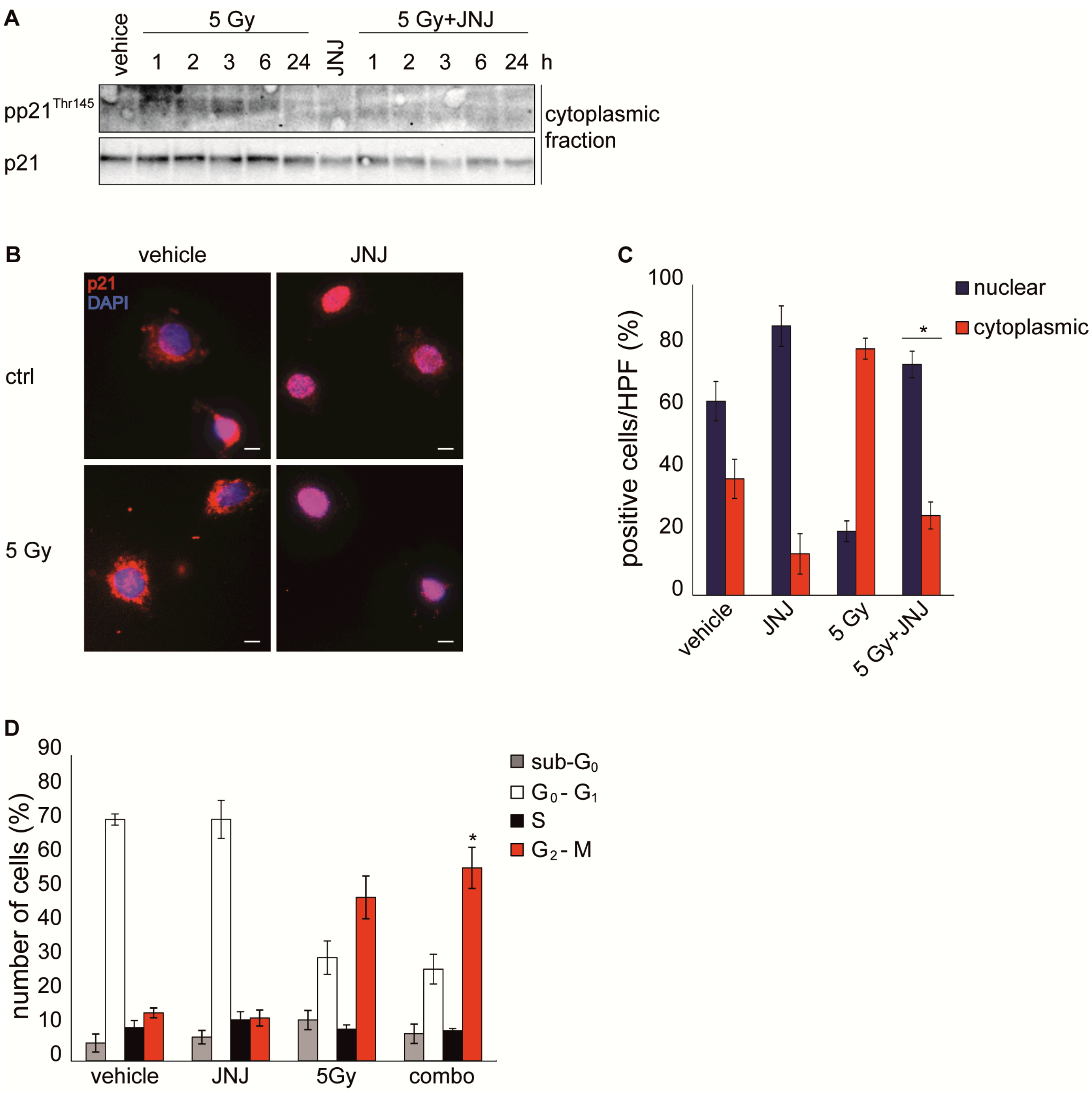

Appendix Fig S7. MET inhibition promotes p21 nuclear translocation.

A, Western blot of BT308 NS showing p21 phosphorylation (pp21^{Thr145}) in cytoplasmic lysates immunoprecipitated with p21 antibodies at the indicated time points after IR in the absence (5 Gy) or in the presence (5 Gy + JNJ) of the MET inhibitor JNJ38877605. B, Representative immunofluorescence staining of p21 (red) on BT205 NS cells 3 h after IR (5 Gy) in the absence (vehicle) or in the presence (JNJ) of JNJ38877605. Ctrl: non-irradiated cells. Nuclei are counterstained with DAPI (blue). Scale bar, 10 μm (63× magnification). C, Quantification of the percentage of cells showing p21 cytoplasmic or nuclear localization in BT205 NS represented in (D) (n = 10 HPF/group). HPF: high-power field. *: *t*-test (5 Gy + JNJ) vs. (ctrl or 5 Gy), P<0.0001.

D, Cell cycle analysis in BT308 NS, 24 h after IR in the absence (5 Gy) or in the presence (combo) of the MET inhibitor JNJ38877605. Data are represented as mean ± SEM. *: *t*-test, $p<0.001$.

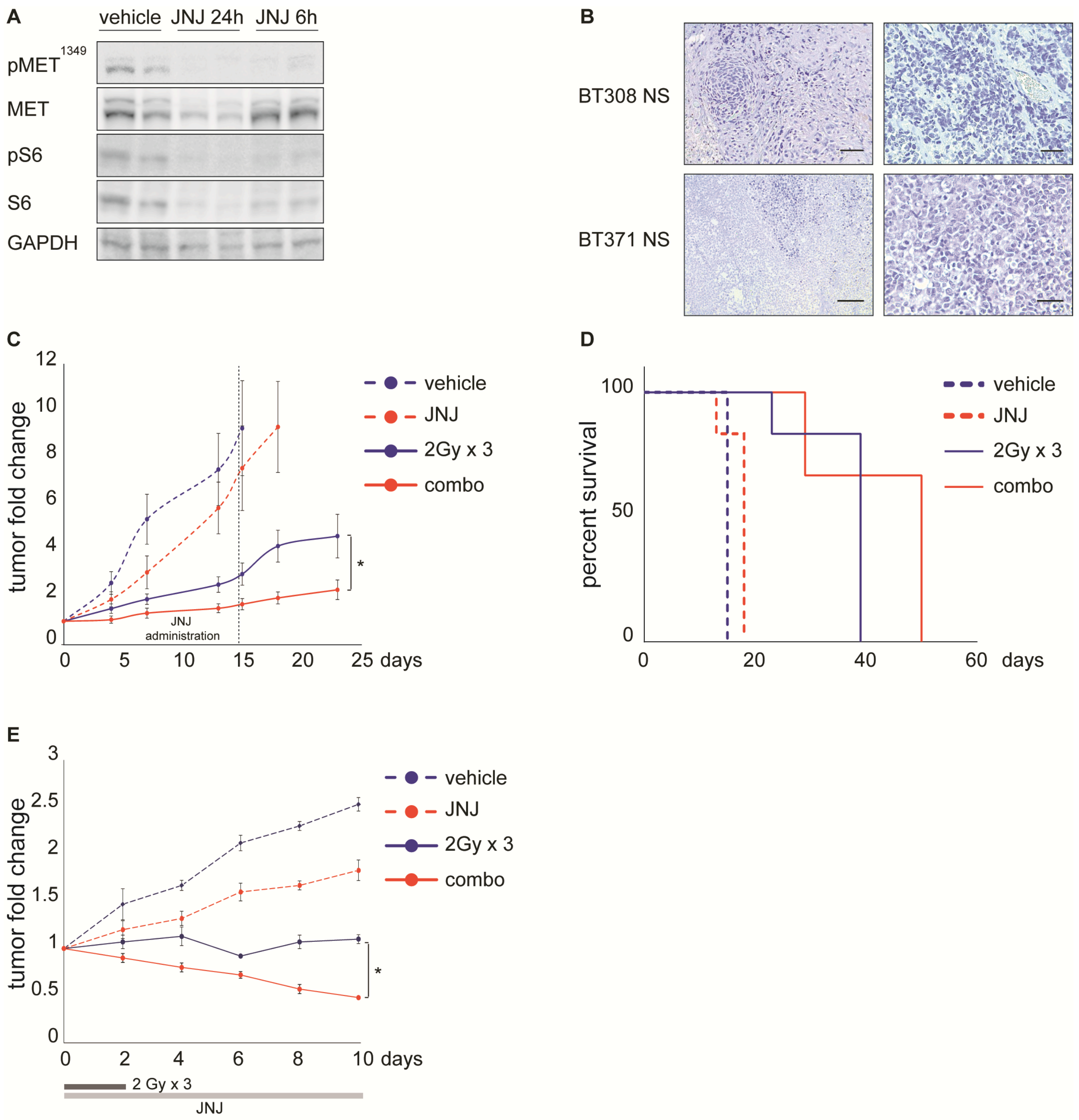

F

cell number	mean tumor volume ± SEM (mm^3)			
	vehicle	JNJ	2 Gy x 3	combo
10^4	1891.5±402.9	1423.98 ±313.5	1198.245±228.2	695.84±170.5
10^3	1753.6±402.6	1192.02±285.9	1959.36±276.9	279.774±39.7
10^2	1132.41±271.9	309.15±101.8	327.07± 180.1	52.305 ±4.2
10^1	171.87± 47.3	63.85±8.14	98.91± 10	32.7

Appendix Fig S8. Met inhibition radiosensitizes GSC-derived GBM and reduces GSC frequency.

A, Western blot showing phosphorylation of MET (pMET^{1349}) and the p70-S6 kinase (pS6) on tumors, generated by intracranial injection of GTL-16 cells, and treated for three consecutive days by oral administration with the MET inhibitor JNJ38877605. Tumors were collected at the indicated time points after the last administration and analyzed. GAPDH was used as loading control. B, Representative images of tumors generated by subcutaneous injection of BT308 and BT371 NS. Scale bar, 100 µm (left, 20× magnification; right, 40× magnification). C, Growth curves of tumors, generated by subcutaneous injection of BT371 NS, irradiated in the absence (2 Gy × 3 days) or in the presence (combo) of JNJ38877605, supplied from day 0 to 15 (n = 6/condition). Vehicle: non-irradiated vehicle-treated tumors. Data are represented as fold change vs. day 0 (mean ± SEM). *: one-way ANOVA test, p<0.05. D, Survival analysis of mice bearing tumors generated and treated as in (C). Log-rank (Mantel-Cox) test, p<0.0001. E, Growth curves of tumors, generated by subcutaneous injection of BT308 NS, irradiated in the absence (2 Gy × 3 days) or in the presence (combo) of JNJ38877605, supplied from day 0 to 10 (n = 3/condition). Vehicle: non-irradiated vehicle-treated tumors. Data are represented as fold change vs. day 0 (mean ± SEM). *: one-way ANOVA test, p<0.05. F, *In vivo* limiting dilution assay: table showing the volume of tumors obtained by injection of the indicated number of cells, derived from tumors treated as indicated. Data are represented as mean ± SEM.

A.

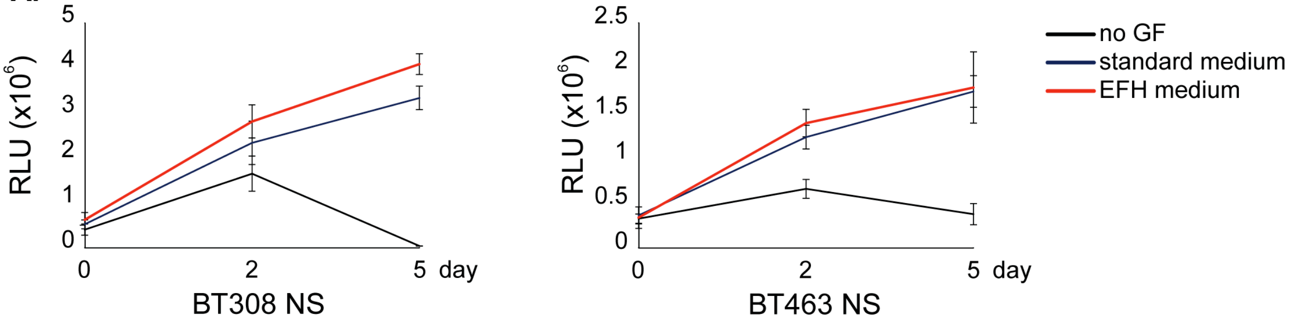

B.

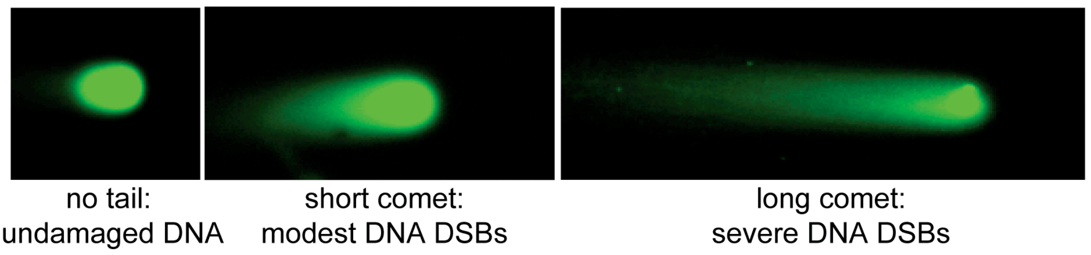

C.

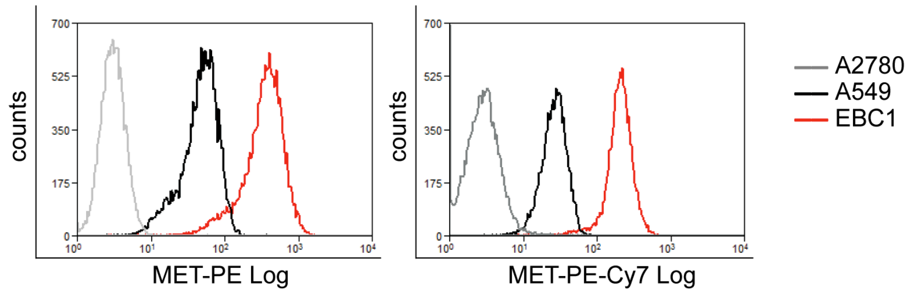

cell lines	MET-PE		MET-PE-Cy7	
	%	MFI	%	MFI
A2780	0.06	21.27	1.03	17.35
A549	96.59	51.26	96.11	27.74
EBC1	99.69	373.72	99.55	203.69

Appendix Fig S9, related to Methods.

A, NS cell proliferation in standard medium (EGF and bFGF) or in a modified medium with lower EGF and bFGF, supplemented with HGF (EFH medium). RLU, relative light units. B, Representative images of comet tails obtained in comet assay on positive control cells (nucleoid with no tail: undamaged DNA; nucleoid with short tail: modest DNA DSBs; nucleoid with long tail: severe DNA DSBs) (63× magnification). C top, Flow cytometric analysis of MET in negative (A2780, grey line), positive (A549, black line) or amplified (EBC1, red line) cell lines, using the commercially available MET-PE antibodies or the in-house conjugated MET-PE-Cy7 antibodies. C bottom, Table showing the percentage and the mean fluorescence intensity (MFI) of MET-expressing cells analyzed with the above antibodies.

Appendix Methods

Neurosphere derivation from human patients and culture

Surgical samples were obtained from consecutive primary GBM provided by the Fondazione IRCCS Istituto Neurologico C. Besta, according to a protocol approved by the institutional Ethical Committee. All Patients signed an informed consent. NS were derived as described (De Bacco *et al.*, 2012), and plated at clonal density in standard NS medium containing human recombinant bFGF (20 ng/ml) and EGF (20 ng/ml). For *in vitro* experiments, NS were kept in a modified medium with a lower concentration of EGF and bFGF (10 ng/ml), and supplemented with human recombinant HGF (10 ng/ml). Both media sustain the same NS proliferation rate (Appendix Fig S9A). NS-derived pseudodifferentiated cells were obtained by culture in medium deprived of growth factors and supplemented with 2% FBS, in pro-adherent culture dishes, for 7 days. GBM cell lines (SNB19, U251 and U87MG), human astrocyte cell lines (NHA, SVG), GTL16, A2780, A549 and EBC1 cell line were kept in culture according to manufacturer's instructions (ATCC). For immortalized cell lines, a passage below 15 was used.

NS irradiation and treatment with inhibitors

NS were irradiated with a 200 kV X-ray blood irradiator (Gilardoni), and a 1 Gy/min dose-rate. In some experiments, HGF (20 ng/ml) was added to standard medium 1 h before irradiation. For MET targeting, the kinase inhibitors JNJ38877605 (500 nM, Janssen Pharmaceutica) (De Bacco *et al.*, 2011), PHA665752 (500 nM; Tocris Cookson Ltd), or Crizotinib (500 nM, Sequoia Research Products) were added to cells 2 h before irradiation; the monovalent monoclonal MET antibody MvDN30 (28µg/ml) was added 24 h before (Pacchiana *et al.*, 2010). Inhibitors for MAPK (PD98059: 20 µM, Calbiochem), AKT (Ly294002: 20 µM, Calbiochem), Aurora Kinase A (MLN8237: 20 µM, Millennium Pharmaceuticals), ATM (CGK733: 10 µM, Sigma) were added 1-4 h before IR. DMSO was used as vehicle for inhibitors, PBS for MvDN30.

Lentiviral transduction of NS

NS were dissociated to single-cell suspensions, and 10^5 cells were transduced in standard medium with lentiviral particles expressing a bicistronic luciferase-GFP construct (CMV-Luc-IRES-GFP, (Vigna *et al.*, 2002) at a Multiplicity Of Infection (MOI) of 5. Transduction efficiency was determined 72 h post-infection by quantifying the amount of GFP-positive cells by FACS analysis (an average of 95% and 99% was obtained).

Xenografts models

All animal procedures were approved by the internal Ethical Committee for Animal Experimentation (FPRC-CESA) and by the Italian Ministry of Health. *Mus musculus*, female NOD.CB17-Prkdc^{scid}/NcrCr mice (Charles River Laboratories) were kept and manipulated under pathogen-free conditions. In details, mice were housed in filtered bottomed, individually sterilized and ventilated cages. Every cage contained a maximum of 6 mice and suitable amounts of sterilized food, water and bedding. Cages were changed weekly to prevent the introduction of minimal-inoculating doses of opportunistic or commensal organisms in the cage environment, and mice were manipulated under a laminar-flow hood.

To generate xenograft models, neurospheres were dissociated into single-cell suspensions, and injected subcutaneously or orthotopically into 6-8 weeks old female NOD-SCID mice.

For subcutaneous transplantation, 2×10^5 cells were resuspended in 100 µl v/v PBS/Matrigel (BD Biosciences) and injected into the right flank. Tumor diameters were measured every 3 days by caliper and tumor volume was calculated using the formula: $4/3\pi \times (d/2)^2 \times (D/2)$, where d and D are the minor and the major tumor axis, respectively. Tumors were established upon reaching an average volume of 400 mm^3.

For orthotopic transplantation, 2.5×10^5 cells expressing the bicistronic luciferase-GFP construct (see above) were resuspended in 2 µl PBS, and delivered into the corpus striatum of the right hemisphere by stereotactic injection (coordinates were as follows: antero-posterior = + 0.8 mm; medio-lateral = + 2.0 mm; dorso-ventral = − 3.0 mm). Intracranial tumor growth was monitored by bioluminescence (BLI) imaging (IVIS® SpectrumCT, Caliper Life Sciences) once a week until animals reached the clinical or experimental endpoints. For BLI, luciferin (firefly D-Luciferin potassium salt, Caliper Life Sciences) was dissolved in PBS (150 mg/kg) and administered to mice by subcutaneous injection. Mice were anesthesized with 2.5% isofluorane in 100% oxygen at a flow rate of 1 L/min. To identify all significant therapeutic responses with a statistical power of 90%, we calculated the mice sample size by considering: (i) volume variations of ±15% as random; (ii) volume variation of at least 20% as a therapeutic effect; (iii) a statistical significance of $p = 0.05$. Accordingly, at least 6 mice/group were used.

Nucleic acid extraction

From NS, nucleic acids were extracted as follows: genomic DNA (gDNA), using the Wizard® Genomic DNA Purification kit (Promega); total RNA, using the mRNeasy Mini kit (Qiagen), according to manufacturer's instructions. Extracted purified nucleic acids were quantified with Nanodrop ND1000 (Thermo Scientific).

Gene sequencing and evaluation of copy number alteration

TP53 and PTEN purified gDNAs were amplified using Platinum® Taq DNA Polymerase (Invitrogen) and specific primer pairs (De Bacco et al., 2012). PCR products were purified using ExoSAP-IT® (Affimetrix) according to manufacturer's instructions. Cycle sequencing was carried out using BigDye Terminator v3.1 Cycle Sequencing kit (Applied Biosystems). Sequencing products were purified using Agencourt CleanSeq (Agencourt Bioscience, Beckman Coulter) and analyzed on a 3730 DNA Analyzer ABI capillary electrophoresis system (Applied Biosystems). Sequences were then analyzed using Chromas Lite 2.01 software (http://www.technelysium.com.au/chromas_lite.html) and compared with reference sequences from the Homo sapiens assembly GRCh37 (February 2009). All identified mutations were than compared with those reported in the Catalogue of Somatic Mutations in Cancer (COSMIC, http://www.sanger.ac.uk/genetics/CGP/cosmic/).

EGFR, PTEN and NFKBIA copy number alterations were assessed on gDNA with real-time PCR, using TaqMan® Copy Number Assays (Applied Biosystems) and the ABI PRISM 7900HT sequence detection system (Applied Biosystems). Relative gene copy number data were calculated by normalizing against endogenous controls (TERT GREB1 and RNaseP). A normal diploid human gDNA was used as calibrator. To discriminate between real EGFR amplification and chr7 polysomy, EGFR copy number was normalized against the copy number of a gene mapped on chr7 and usually not amplified (HGF), and defined amplified when EGFR/HGF copy number was > 5. Reported values are the mean ± SEM of two independent experiments in triplicate.

Microarray analysis

Gene expression profiling and NS classification according to Verhaak's subtypes (Verhaak *et al*., 2010) was performed as previously described (De Bacco *et al*., 2012).

Real-Time PCR

500 ng of purified mRNA was reverse-transcribed using High Capacity Reverse Transcriptase kit (Applied Biosystem) according to manufacturer's instructions. Real-time PCR was performed using commercially available primers and probe sets for MET, RAD51, and SOX2 with TaqMan PCR Master Mix (Applied Biosystems), and ABI PRISM 7900HT sequence detection system. Expression levels were normalized against endogenous controls (β2 microglobulin or β-actin) and non-treated cells were used as calibrator. Reported values are the mean ± SEM of at least two independent experiments in triplicate.

Comet assay

To evaluate DNA double strand breaks (DSBs) induced by IR, Neutral Comet Assay (Trevigen) was used according to manufacturer's instructions. For each experimental point, 103 cells were used. Comet slides were examined by eye at 10× magnification under a fluorescence microscope. Measurements of DNA damage was evaluated semi-quantitatively using the following definitions: nucleoid with no tail: undamaged DNA; nucleoid with short tail (short comet): modest DNA DSBs; nucleoid with long tail (long comet): severe DNA DSBs (representative images of nucleoid with no tail, and short and long comet were shown in Appendix Fig S9B). Experiments were repeated at least twice in duplicate, and a representative experiment was shown.

Western blotting

Protein expression and phosphorylation were analyzed in whole-cell lysates solubilized in boiling Laemmli buffer, or in cytoplasmic and nuclear portions fractionated as previously described (De Bacco *et al*., 2011), or in snap-frozen and mechanically dissociated xenograft tissues, solubilized in RIPA buffer. Equal amounts of proteins were resolved by SDS-PAGE in reducing conditions and analyzed by western blotting with the following antibodies: rabbit polyclonal anti-MET (C12; #SC-10, Santa Cruz Biotechnology), anti-AKT (#BK9272), anti-phospho-AKT (#BK4060), anti-phospho-Chk2 (#BK2661), anti-p44/42 MAP Kinases (#BK9102), anti-phospho-p44/42 MAP Kinases (#BK9101), anti-phospho-p70 S6 kinase (#BK4858), anti-p70 S6 kinase (#BK9202), anti-RAD51 (#BK8875), anti-phospho H2AX (#BK9718), anti-phospho Aurora A/B/C (#BK2914), anti-Aurora A (#BK14475), anti-cleaved caspase 3 (#BK9664), anti-cleaved PARP (#BK9546) and anti-ATM (#BK2873) (Cell Signaling Technology), and anti-phospho MET (Tyr 1349) (#04-1063, Merck Millipore), and mouse-monoclonal anti-phospho ATM (#ab1292, Abcam). For control of equal amount of sample loading, rabbit polyclonal anti-β actin (#8457), anti-GAPDH (#5174), anti-H3 (#4499) (Cell Signaling Technology), anti-laminB (#BKNA12, Merck Millipore), or anti-vinculin (#V9131, Sigma) antibodies were used. For MET and p21 phosphorylation analysis, equal amounts of proteins were immunoprecipitated using rabbit polyclonal anti-MET (C12) or anti-p21 (#BK2947, Cell Signaling Technology) respectively, and analyzed by western blotting with mouse monoclonal anti-phospho Tyr (clone 4G10; #05-321, Merck Millipore) or rabbit polyclonal anti-phospho p21 (Thr 145; #SC-20220, Santa Cruz Biotechnology) antibodies. Antibodies were visualized with appropriate horseradish peroxidase-conjugated secondary antibodies (Amersham), and the enhanced chemiluminescence system (Promega). Blot images were captured using a molecular imager (GelDoc XR; Bio-Rad Laboratories) and are representative of results obtained in

at least two independent experiments. Densitometric analysis was performed using NIH ImageJ software (National Institutes of Health, Bethesda, MD).

Immunophenotypical analysis and Fluorescence-Activated Cell Sorting

NS were dissociated and resuspended in PBS 3% BSA at a concentration of 2×10^5 cells/100 µl. The following antibodies were used: phospho-H2AX-FITC (Ser139; #17-344, Merck Millipore); Olig2-Alexa-Fluor 488 (#MABN50A4, Merck Millipore); HGF R/c-MET (clone 95106; MAB3582, R&D System), conjugated by the manufacturer with PE or APC, or in house with PE-Cy7, according to manufacturer's instructions (R&D System). The three antibodies display comparable specificity (Appendix Fig S9C). The % of positive cells was calculated by comparing unstained vs. stained cells in each sample, as to avoid bias associated with morphological, and ensuing autofluorescent, changes due to irradiation (Maecker and Trotter, 2006). Before analysis, DAPI (Roche) was added to exclude dead cells. Analysis was performed on a CyAn ADP (Beckman Coulter) equipped with 488 nM, 405 nM and 642 nM solid state lasers. Data were collected and processed using Summit 4.3 software (Beckman Coulter). For Fluorescence-Activated Cell Sorting, dissociated cells were stained with anti-MET-PE antibodies, and filtered with Filcons filters (50 µm, BD Biosciences) to avoid aggregates. Cell sorting was performed with a MoFlo™ XDP nine-color cell sorter (Beckman Coulter). Experiments were repeated at least twice, and a representative experiment was shown.

PKH-26 staining

Dissociated NS cells were stained with PKH-26 dye (1:2000, Sigma), according to manufacturer's instructions, and plated at clonal density. Derived NS were passaged for 2 weeks to allow adequate PKH-26 dilution before treatments. Percentage of PKH-26 positive cells was evaluated by flow cytometry as above. Experiments were repeated at least twice, and a representative experiment was shown.

Annexin V evaluation

Annexin V Apoptosis Detection Kit (BD Pharmingen™) was used according to manufacturer's instructions for apoptosis evaluation by flow cytometry. To discriminate apoptosis in MET^{high} and MET^{neg} subpopulation, cells were incubated with anti-MET-APC as above and washed twice with Binding Buffer prior to Annexin V staining. Reported values are the mean ± SEM of two independent experiments.

Cell cycle analysis

Cell cycle was analyzed according to standard procedures. Briefly, 10^6 cells were fixed with 70% Ethanol overnight at -20°C, stained with propidium iodide (50 µg/ml) in RNaseA solution (DNAcon3 kit ConsulTS) for 3 h at 4°C in the dark, and analyzed by flow-cytometry as above. Data are the mean ± SEM of at least two independent experiments.

Cell viability and caspase activation assays

To assess NS viability and apoptosis, ATP cell content and Caspase 3/7 activity were measured using Cell Titer Glo® and Caspase-Glo® 3/7 Assay (Promega) respectively, according to manufacturer's instructions. Cells were plated at clonal density (10 cells/µl) in 96-well plates, and treated as described 24 h after seeding (day 0). Cell viability and caspase 3/7 activity were measured using a GloMax 96 Microplate Luminometer (Promega). Data are reported with respect to non-treated cells as mean ± SEM of at least two independent experiments in quadruplicate.

Radiobiological clonogenic assay

Met^{high} and Met^{neg} cell subpopulations were sorted as described above and seeded as single cell/well. 24 h after sorting, cells were irradiated in the presence or in the absence of MET inhibitors, and then cultured for 14 days. The number of growing NS was counted, and the surviving fraction was calculated using the formula, modified from (Franken *et al.*, 2006): [(n° of NS formed after treatment) / (PE)] x 100, where PE (plating efficiency) is the percentage of NS in control conditions. The reported values are the mean ± SEM of two independent experiments.

Limiting dilution sphere forming assay *in vitro*

Limiting dilution sphere forming assay was performed to assess the frequency of GSC in NS established from original tumors, or in cells freshly dissociated from xenografts obtained by NS transplantation. Viable cells (trypan blue exclusion test) were plated into 96-well plates at decreasing concentrations (ranging from 150 cells/well to 1 cell/well). The number of growing NS was counted 14-21 days after seeding. Data were evaluated through the ELDA software (http://bioinf.wehi.edu.au/software/elda/) (Hu *et al.*, 2009), and reported as percentage of stem-like cells ± CI.

Serial transplantation *assay*

NS were irradiated *in vitro* (p0) as described above, and 24 h after irradiation 10^3 cells were resuspended in 100 µl v/v PBS/Matrigel, and subcutaneously injected in 4 mice (p1). Tumor

formation was monitored once a week by caliper as described above. From p1 tumors, cells were dissociated as described above for human tumor specimens, and, when NS formed, 10^3 cells in 100 µl v/v PBS/Matrigel were subcutaneously injected in 6 mice (p2). From p2 tumors, cell isolation and NS formation was repeated as above, and cells were assessed by *in vivo* limiting dilution assay, by injecting 10, 10^2, 10^3, or 10^4 cells in 6 mice per condition (p3). Frequency of stem-like cells was calculated using ELDA software as above and reported as stem-like cells per 10,000 cells.

Limiting dilution assay *in vivo*

In vivo serial dilution tumor-propagating assay was performed to evaluate the relative frequency of GSCs in subcutaneous xenografts after radiosensitization treatment. Subcutaneous xenografts were obtained as described above. Mice were randomized into four groups: (i) vehicle treated; (ii) treated with IR (2 Gy × 3 consecutive days); (iii) treated with JNJ38877605 (50 mg/kg, by daily oral gavage); (iv) treated with association of JNJ38877605 and IR as above (combo). The inhibitor JNJ-38877605 was administered starting from the day before irradiation (day -1) and prolonged up to day 10 in which 50% of tumor volume regression was reached in the combo group, as compared to day 0. At this point, tumors from each group were explanted and dissociated to re-derive tumor cells immediately cultured in stem conditions as described above. After cell recovery (about 10 days), single-cell suspensions of viable tumor-derived cells for each group (trypan blue exclusion test) were injected in the right flank of NOD-SCID mice at the following dilution dose 10, 10^2, 10^3 or 10^4 in 100 µl v/v PBS/Matrigel (6 mice/condition). The frequency of stem-like tumor propagating cells within heterogeneous cell populations was evaluate on the efficiency of secondary xenograft formation and data were evaluated through the ELDA software as above and reported as stem-like cells per 10,000 cells.

Irradiation and radiosensitization of xenograft models

To establish treatment groups and to minimize the effect of subjective bias, mice with either subcutaneous tumors of approximately 400 mm^3, or orthotopic tumors with a BLI average radiance signal of 7×10^5 p/s/sr/cm^2, were randomized and group allocated by LAS software (Baralis *et al.*, 2012). During randomization and generation of the experimental arms, mice were excluded from the studies if the tumor volume was indicated as outlier by LAS software. No tumor formation, due to technical problems during neurosphere injections, was also considered as a criterion of exclusion. Xenografted mice were anesthetized by i.p. injection (zoletil 40 mg/kg + xylazine 7.5 mg/kg) and placed in a plexiglass pie cage (2Biological Instruments). Two days before irradiation, high-resolution CT scans (Toshiba Aquilion LB) were acquired to delineate the Planning Target Volume

(PTV). For subcutaneous xenografts, lungs and gastro-intestinal tract were considered organs at risk (OARs). For intracranial xenografts, the PTV corresponded to whole mouse head (no OARs exclusion). A total dose of 6 Gy was delivered in 3 consecutive fractions (day 0, 1, 2) with TomoTherapy HD or the equivalent Hi-Art (Accuray, Inc.). TomoDirect Intensity Modulated Radiation Therapy (IMRT) technique was applied, allowing the delivery of 95% of the prescribed dose to PTV, while sparing OARs. In order to further optimize dose delivery, a bolus layer (ELASTO-GEL) was placed upon mice's body for subcutaneous, and upon mice’s head for intracranial tumors. Before the delivery of each IR dose, a Megavoltage Computed Tomography was performed in order to verify the correct mouse positioning. After irradiation, mice were awakened on a heating bed and housed in their cages.

For radiosensitization experiments, mice were randomized into four groups: (i) vehicle treated; (ii) treated with IR (2 Gy × 3 consecutive days); (iii) treated with JNJ38877605 (50 mg/kg, by daily oral gavage); (iv) treated with association of JNJ38877605 and IR as above (combo). In the latter case, JNJ-38877605 was administered starting from the day before irradiation (day -1), up to 15 or 30 days after irradiation in subcutaneous or orthotopic xenografts, respectively. Mice were monitored daily and, in case of systemic suffering, neurological symptoms or significant loss of body weight, were sacrificed by intraperitoneal (i.p.) anesthesia (zoletil 40 mg/kg + xylazine 7.5 mg/kg) followed by CO_2 inhalation. In survival experiments (subcutaneous xenografts) mice were sacrificed when tumor size reached the pre-established clinical endpoint of 1600 mm^3; animals euthanized before reaching the clinical endpoint were included in survival curve as censored observations.

In the orthotopic model, mice were euthanized at day 62 (at the onset of neurological symptoms in the control group). Explanted brains were immediately analyzed for GFP signal detection (e_x 465 nm, e_m 520 nm; IVIS® SpectrumCT). Data were collected by A.D.A. with no blinding.

Immunofluorescence analysis

Dissociated neurospheres were seeded on poly-lysine coated slides, treated as indicated, fixed and incubated with rabbit polyclonal anti-p21 (1:50 in PBS/1% BSA, Cell Signaling Technology) antibodies. Xenograft specimens were embedded in OCT, cryo-sectioned at 10 μm, and incubated with mouse monoclonal anti-Met DO-24 antibodies (1:100 in PBS/1% BSA) (Prat *et al.*, 1998). Anti-mouse Alexa-Fluor 555 (1:1000, Molecular Probes by Life Technologies) was used as secondary antibody. Nuclei were counter-stained with DAPI, according to standard protocols. Images were acquired with Leica DMI 3000 B or Leica TCS SP2 AOBS confocal laser-scanning microscope (Leica). Images have been analyzed with NIH ImageJ software (National Institutes of

Health, Bethesda, MD); the Relative Fluorescence Intensity was estimated with Leica LAS AF lite software. For analysis of cytoplasmic/nuclear localization of p21 in irradiated MET-pos-NS, at least 10 HPF (63 × magnification) were evaluated; for MET quantification, 6 HPF (63 × magnification) in two sections from each tumor, for a total of 3 mice/group, were counted.

Association of MET expression with disease free and overall survival in public dataset

MET mRNA expression data were obtained from the public TCGA GBM dataset available on cBioPortal (http://www.cbioportal.org), selecting the provisional dataset (June 2015, Agilent microarray data, n = 401), and applying an mRNA Z-score threshold of ± 2. Disease-free and overall survival curves were obtained with the cBioPortal software using the Kaplan–Meier method (Cerami *et al.*, 2012; Gao *et al.*, 2013).

Collection of matched primary and recurrent human GBMs.

Surgical samples were derived from primary and matched recurrent GBMs surgically removed at the Spedali Civili di Brescia, according to a protocol approved by the institutional Ethical Committee. All Patients signed an informed consent. Patients were treated with standard fractionated radiotherapy (60 Gy) and concomitant chemotherapy (75 mg/m^2 of Temozolomide) on daily basis for 6 weeks, and then with Temozolomide alone (150-200 mg/m^2) for 5 days in a 28-day for 6 cycles, and up to 12 cycles, if no treatment related adverse events were noted or there was no evidence of tumor progression based on both clinical evaluation and MRI findings. At tumor progression, second surgery was performed, and a second line treatment was offered to selected Patients (Fotemustine).

Immunohistochemical staining of MET on matched primary/recurrent GBMs.

MET immunohistochemical staining were performed on representative paraffin embedded sections (2 µm thick), incubated with rabbit polyclonal anti-Met C12 (1:50 in TBS/1% BSA, Santa Cruz Biotechnology), after blocking of endogenous peroxidase activity with 0.3% H_2O_2 in methanol and antigen retrieval in 1mM Citrate buffer (pH 6.0) in a thermostatic bath. Signal was revealed using the NovoLinkTM Polymer Detection System (NovocastraTM), followed by Diaminobenzydine (DAB) as chromogen and Hematoxylin as counterstain. Images were acquired with an Olympus Bx60 microscope equipped with a DP70 camera and CellF imaging software (Soft Imaging System GmbH). Expression of MET was evaluated by P.L.P. and M.C., and was scored semi-quantitatively as percentage of positive cells and staining intensity. For the percentage of positive immunoreactive cells, the following scores were used: 0, 0-5%; 1, 6-29%; 2, 30-69%; and 3, 70-100%. The staining

intensity was evaluated as follows: 0, negative staining; 1, low intensity; 2, moderate intensity; 3, strong intensity. The cumulative score (from 0 to 6) was obtained by adding the positivity score (percentage of positive immunoreactive cells) and the intensity score (staining intensity of cells).

Statistical analysis.

Values were expressed as mean ± standard error of the mean (SEM). *In vitro* experiments were repeated at least twice in quadruplicate. Statistical analyses were performed using GraphPad Prism Software (GraphPad Software Inc.). Unpaired two-sided Student *t*-test, one-way Analysis of Variance (ANOVA) or Wilcoxon test were used as indicated. Survival curves were analyzed using the Kaplan-Meier method with groups compared by respective median survival; log-rank P value was measured using the Mantel-Cox test. Chi-squared tests and confidence intervals 95% (CI 95%) for limiting dilution assays were performed by ELDA software. A p-value < 0.05 was considered to be significant for all the statistical tests used.

Appendix References

Baralis E, Bertotti A, Fiori A, Grand A. LAS: a software platform to support oncological data management. J Med Syst 2012;36 Suppl 1:S81-S90.

Cerami E, Gao J, Dogrusoz U, Gross BE, Sumer SO, Aksoy BA et al. The cBio cancer genomics portal: an open platform for exploring multidimensional cancer genomics data. Cancer Discov 2012;2:401-4.

De Bacco F, Casanova E, Medico E, Pellegatta S, Orzan F, Albano R et al. The MET Oncogene Is a Functional Marker of a Glioblastoma Stem Cell Subtype. Cancer Res 2012;72:4537-50.

De Bacco F, Luraghi P, Medico E, Reato G, Girolami F, Perera T et al. Induction of MET by ionizing radiation and its role in radioresistance and invasive growth of cancer. J Natl Cancer Inst 2011;103:645-61.

Franken NA, Rodermond HM, Stap J, Haveman J, van BC. Clonogenic assay of cells in vitro. Nat Protoc 2006;1:2315-9.

Gao J, Aksoy BA, Dogrusoz U, Dresdner G, Gross B, Sumer SO et al. Integrative analysis of complex cancer genomics and clinical profiles using the cBioPortal. Sci Signal 2013;6:l1.

Hu Y, Smyth GK. ELDA: extreme limiting dilution analysis for comparing depleted and enriched populations in stem cell and other assays. J Immunol Methods 2009;347:70-8.

Maecker HT and Trotter J. Flow Cytometry Controls, Instrument Setup, and the Determination of Positivity. Cytometry Part A 2006; 69A:1037-1042.

Pacchiana G, Chiriaco C, Stella MC, Petronzelli F, De SR, Galluzzo M, Carminati P, Comoglio PM, Michieli P, and Vigna E (2010) Monovalency unleashes the full therapeutic potential of the DN-30 anti-Met antibody. J Biol Chem 285: 36149-36157

Prat M, Crepaldi T, Pennacchietti S, Bussolino F, Comoglio PM. Agonistic monoclonal antibodies against the Met receptor dissect the biological responses to HGF. J Cell Sci 1998;111 (Pt 2):237-47.

Verhaak RG, Hoadley KA, Purdom E, Wang V, Qi Y, Wilkerson MD et al. Integrated genomic analysis identifies clinically relevant subtypes of glioblastoma characterized by abnormalities in PDGFRA, IDH1, EGFR, and NF1. Cancer Cell 2010;17:98-110.

Vigna E, Cavalieri S, Ailles L, Geuna M, Loew R, Bujard H et al. Robust and efficient regulation of transgene expression in vivo by improved tetracycline-dependent lentiviral vectors. Mol Ther 2002;5:252-61.
