# Supplementary material for: MET inhibition overcomes radiation resistance of glioblastoma stem‐like cells
Source: EMBO Mol Med. 2016 Apr 4;8(5):550–68. doi: 10.15252/emmm.201505890 (PMC5130292; doi:10.15252/emmm.201505890)
Supplement: Supplementary file 2 — Expanded View Figures PDF [file EMMM-8-550-s002.pdf]

Expanded View Figures

Figure EV1. GSCs are radioresistant regardless of genetic and phenotypic features.

A Cell viability of NS and astrocyte cell lines measured at the indicated time points after irradiation (2 Gy) (fold versus non-irradiated cells, ctrl). Data are represented as mean ± SEM. *: one-way ANOVA, $P < 0.05$. $n = 2$.

B, C Box plots representing NS viability measured 72 h after irradiation (2–10 Gy) with respect to the most frequent GBM genetic alterations (B) or transcriptional profiles (C). Data are represented as percentage of viable cells versus non-irradiated cells (wt: wild-type; amp: gene copy number difference between EGFR and HGF > 5; loss: PTEN biallelic inactivation by deletion and/or mutation; mut: mutated TP53; del: NFKBIA gene copy number < 1.5; CL: classical subtype; MES: mesenchymal subtype; PN: proneural subtype). No statistically significant difference (one-way ANOVA) has been found among genetically or phenotypically different groups. Data information for box plots: median (line), upper/lower quartile (box), min/max (error bars).

© 2016 The Authors

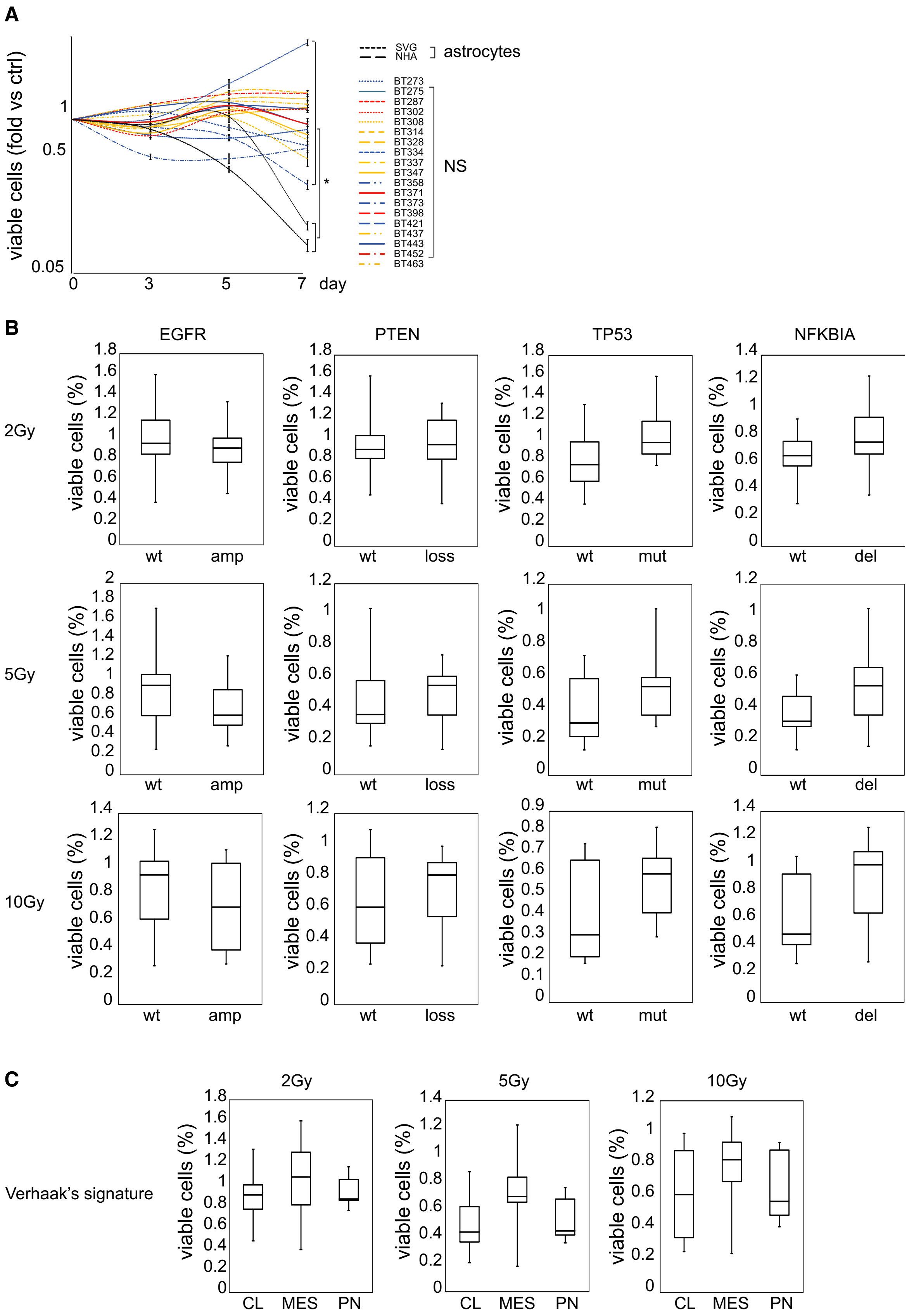
A
viable cells (fold vs ctrl)
1
0.5
0.05
0
3
5
7
day
SVG
NHA
astrocytes
BT273
BT275
BT287
BT302
BT308
BT314
BT328
BT334
BT337
BT347
BT358
BT371
BT373
BT398
BT421
BT437
BT443
BT452
BT463
NS
*
B
EGFR
PTEN
TP53
NFKBIA
2Gy
5Gy
10Gy
viable cells (%)
wt
amp
loss
mut
del
C
Verhaak's signature
2Gy
5Gy
10Gy
CL
MES
PN

Figure EV1.

A

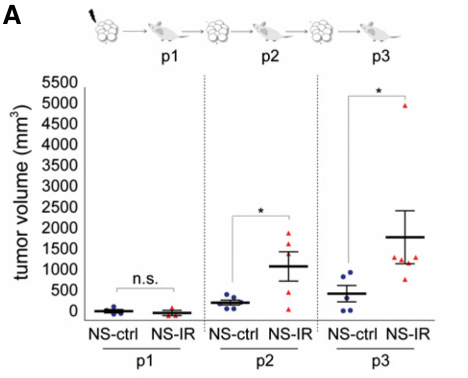

B

treatment		tumor take	tumor volume (mm³)
p1	NS-ctrl	4/4	122.39 ± 41.2
	NS-IR	3/4	79.41 ± 62.9
p2	NS-ctrl	6/6	329.01 ± 57.3
	NS-IR	5/6	1200.8 ± 352.8
p3	NS-ctrl	5/6	543.58 ± 196.5
	NS-IR	6/6	1905.2 ± 637.4

Figure EV2. Increased tumorigenesis in serial passages of irradiated NS.

A Top: schematic representation of serial xenotransplantation. Bottom: scatter plot showing take and volume (14 weeks after cell injection) of tumors generated by control (NS-ctrl) and irradiated (NS-IR) NS for each transplantation passage (10^3 cells). *: *t*-test, $P < 0.05$; n.s. not significant. $n = 4$ for p1; $n = 6$ for p2 and p3.

B Table showing data represented in (A).

Data information: Data are mean ± SEM.

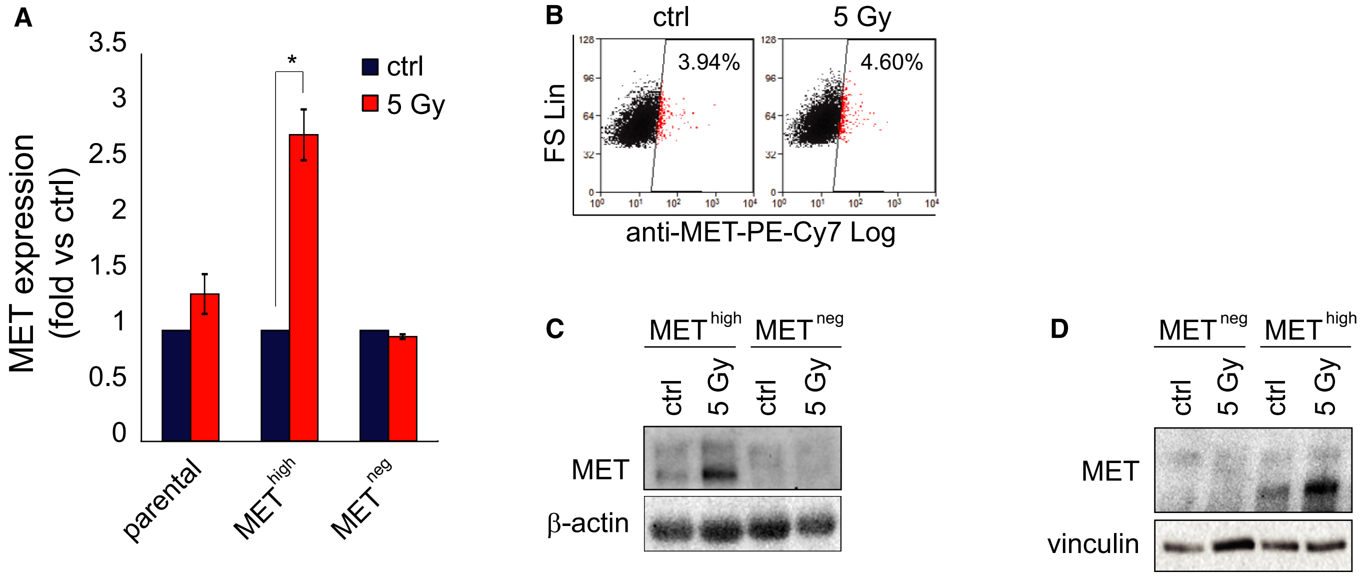

Figure EV3. MET is upregulated in sorted MET^{high} but not MET^{neg} NS subpopulations.

A qPCR of MET expression in parental BT308NS and sorted MET^{high} and MET^{neg} subpopulation 24 h after IR (5 Gy, fold versus non-irradiated cells, ctrl). *: *t*-test, $P < 0.02$. $n = 3$.

B Flow cytometric analysis of MET in the MET^{neg} subpopulation sorted from BT308NS, 24 h after IR (5 Gy). The percentage of MET-expressing cells is indicated. Ctrl: non-irradiated cells.

C, D Western blot showing MET protein expression in MET^{high} and MET^{neg} subpopulations sorted from BT452NS (C) or BT308NS (D) 24 h after treatment (5 Gy). Vinculin and β-actin were used as loading controls. Ctrl: non-irradiated cells.

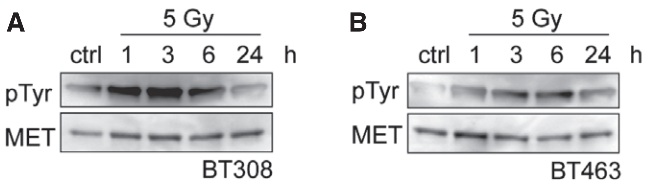

Figure EV4. MET is hyperphosphorylated after NS irradiation.

A, B Western blots of BT308NS (A) and BT463NS (B) showing MET tyrosine phosphorylation (pTyr) in NS kept in the presence of HGF (10 ng/ml), extracted at the indicated time points after irradiation (5 Gy), and immunoprecipitated with MET antibodies.

 © 2016 The Authors
