# Supplementary figures and images for: MET inhibition overcomes radiation resistance of glioblastoma stem‐like cells
Source: EMBO Mol Med. 2016 Apr 4;8(5):550–68. doi: 10.15252/emmm.201505890 (PMC5130292; doi:10.15252/emmm.201505890)

Figure 4B

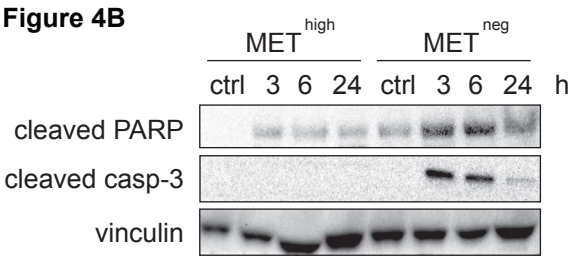

Original scans Figure 4B

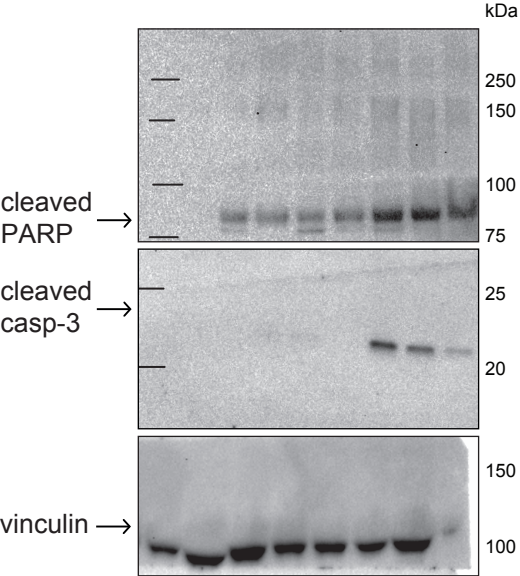

Figure 4F

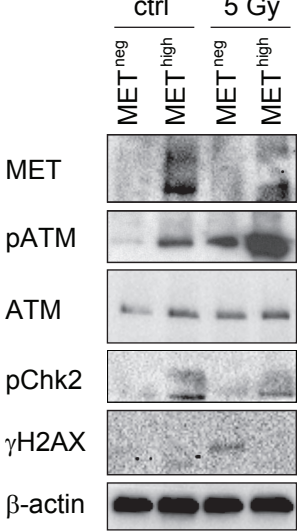

Original scans Figure 4F

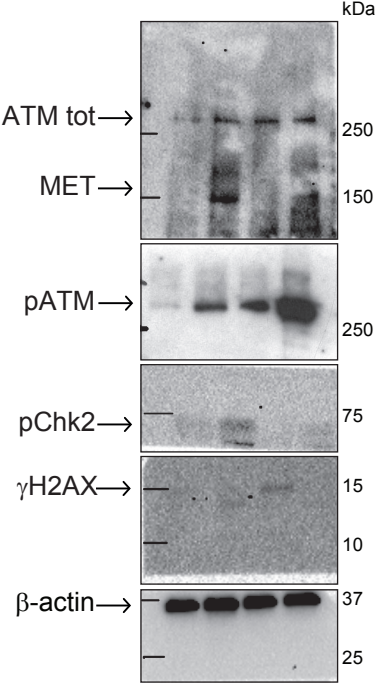

Supplement: Supplementary file 4 — Source Data for Figure 4 [file EMMM-8-550-s003.pdf]

Figure 5F

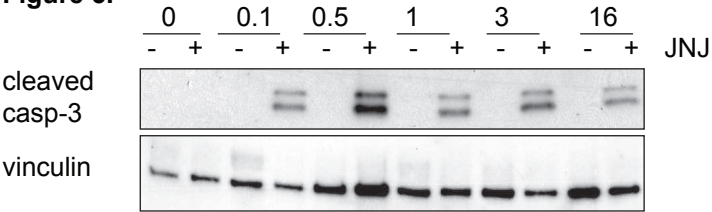

Original scans Figure 5F

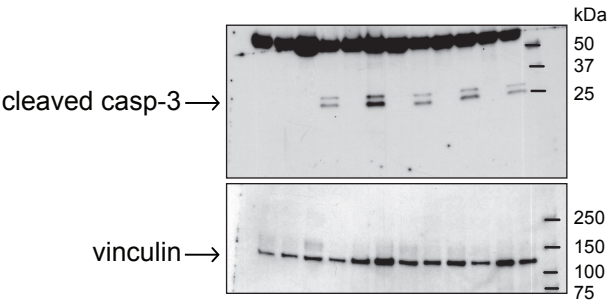

Supplement: Supplementary file 5 — Source Data for Figure 5 [file EMMM-8-550-s004.pdf]
