# Supplementary material for: MET inhibition overcomes radiation resistance of glioblastoma stem‐like cells
Source: EMBO Mol Med. 2016 Apr 4;8(5):550–68. doi: 10.15252/emmm.201505890 (PMC5130292; doi:10.15252/emmm.201505890)
Supplement: Supplementary file 6 — Source Data for Figure 6 [file EMMM-8-550-s005.pdf]

Figure 6A

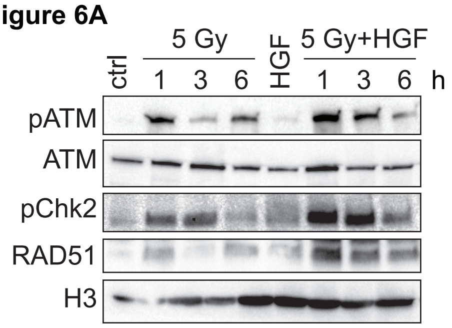

Original scans Figure 6A

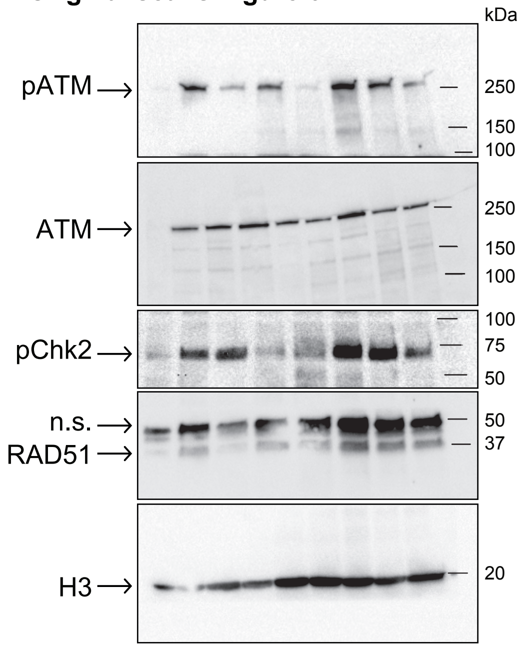

Figure 6B

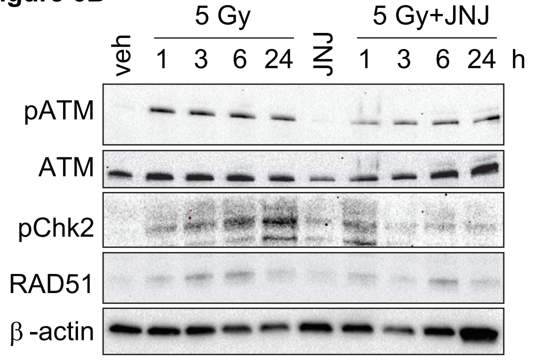

Original scans Figure 6B

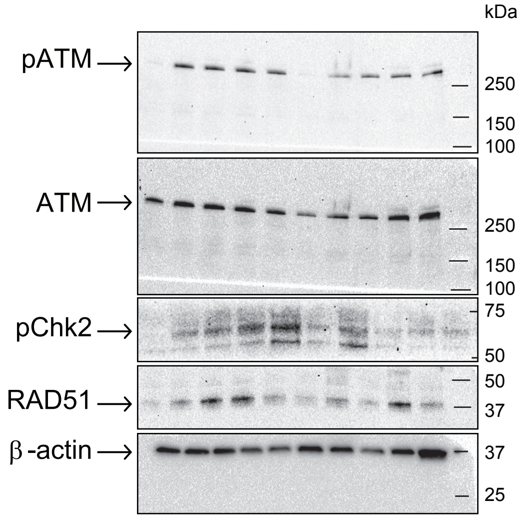

Figure 6D

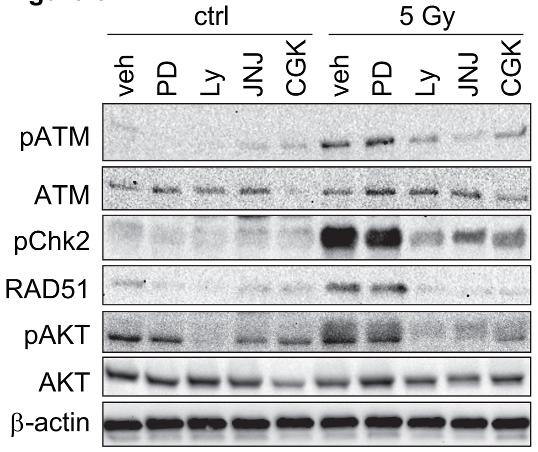

Original scans Figure 6D

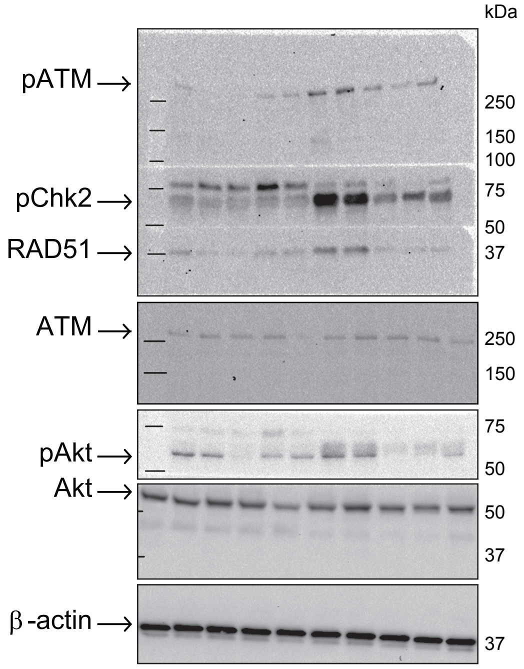

Figure 6E

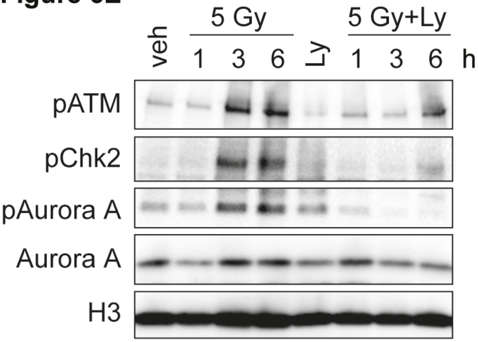

Original scans Figure 6E

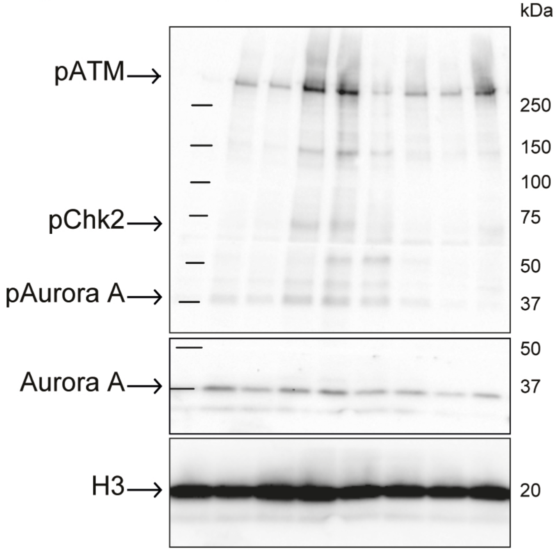

Figure 6F

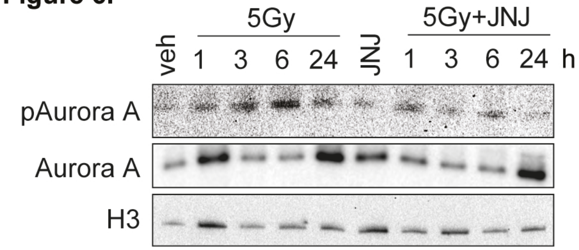

Original scans Figure 6F

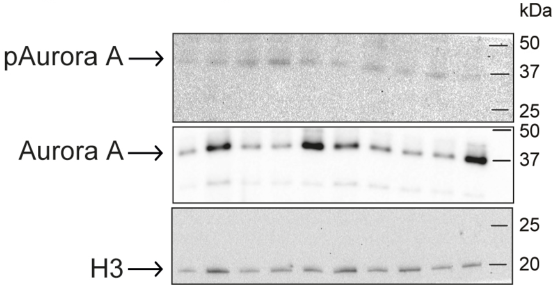

Figure 6G

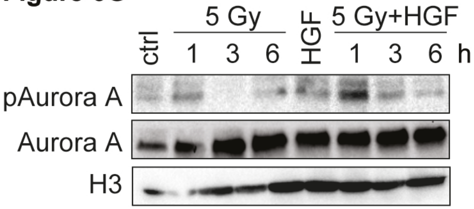

Original scans Figure 6G

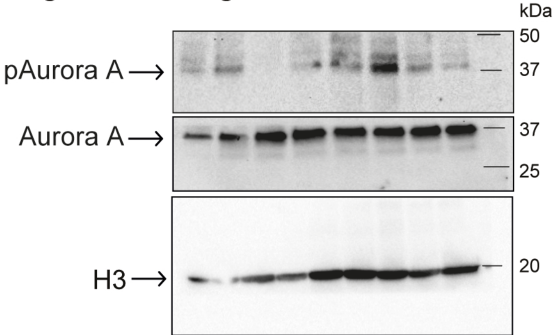

NB. the same H3 blot has been shown for Figure 6A and 6G as they are part of the same experiment
