# Supplementary material for: MET inhibition overcomes radiation resistance of glioblastoma stem‐like cells
Source: EMBO Mol Med. 2016 Apr 4;8(5):550–68. doi: 10.15252/emmm.201505890 (PMC5130292; doi:10.15252/emmm.201505890)
Supplement: Supplementary file 7 — Source Data for Figure 7 [file EMMM-8-550-s006.pdf]

Figure 7C

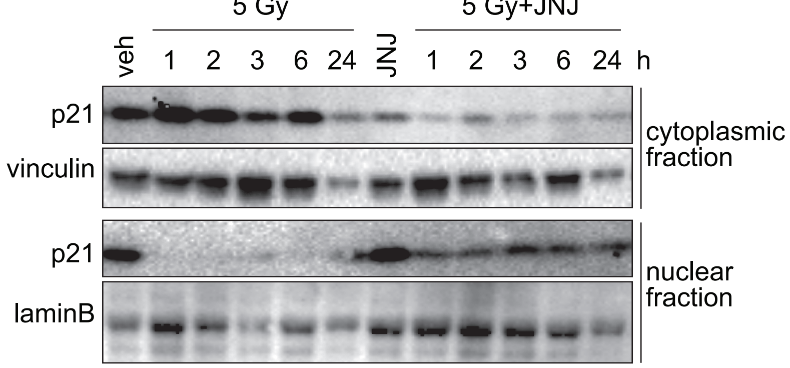

Original scans Figure 7C

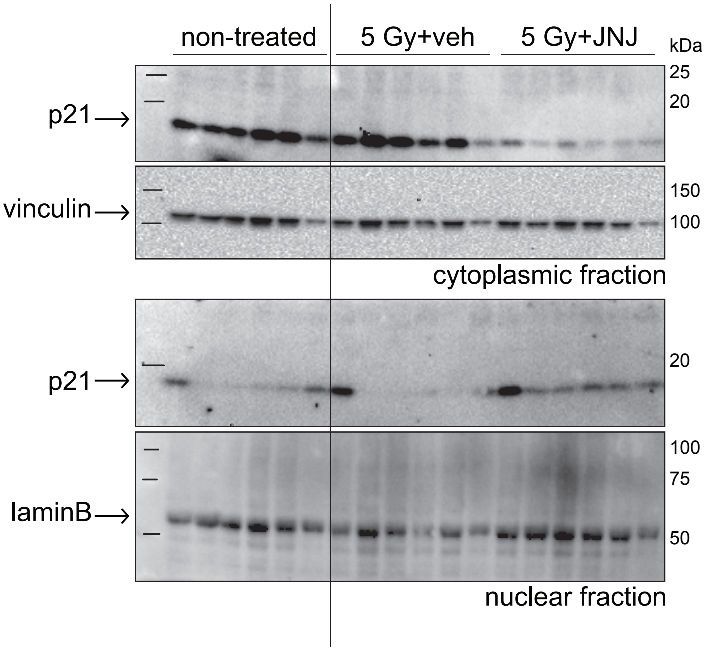

NB. lanes 1-6 are multiple control samples (irradiated cells without vehicle)
